# Varying sex and identity of faces affects face categorization differently in humans and computational models

**DOI:** 10.1038/s41598-023-43169-9

**Published:** 2023-09-26

**Authors:** Isabelle Bülthoff, Laura Manno, Mintao Zhao

**Affiliations:** 1 Max Planck Institute for Biological Cybernetics, Tübingen, Germany; 2 University of East Anglia, Norwich, UK

**Keywords:** Neuroscience, Psychology

## Abstract

Our faces display socially important sex and identity information. How perceptually independent are these facial characteristics? Here, we used a sex categorization task to investigate how changing faces in terms of either their sex or identity affects sex categorization of those faces, whether these manipulations affect sex categorization similarly when the original faces were personally familiar or unknown, and, whether computational models trained for sex classification respond similarly to human observers. Our results show that varying faces along either sex or identity dimension affects their sex categorization. When the sex was swapped (e.g., female faces became male looking, Experiment 1), sex categorization performance was different from that with the original unchanged faces, and significantly more so for people who were familiar with the original faces than those who were not. When the identity of the faces was manipulated by caricaturing or anti-caricaturing them (these manipulations either augment or diminish idiosyncratic facial information, Experiment 2), sex categorization performance to caricatured, original, and anti-caricatured faces increased in that order, independently of face familiarity. Moreover, our face manipulations showed different effects upon computational models trained for sex classification and elicited different patterns of responses in humans and computational models. These results not only support the notion that the sex and identity of faces are processed integratively by human observers but also demonstrate that computational models of face categorization may not capture key characteristics of human face categorization.

## Introduction

For humans, faces are fascinating objects that attract their attention. When we see a face, we are immediately aware of the person’s sex, age or mood, and whether this is the face of a friend or of a stranger. The mechanisms underlying the seemingly automatic processing of faces in relation to their identity and sex have been heavily investigated in previous studies^1–3^. In an influential face recognition model, Bruce and Young^4^ propose that face familiarity and sex classification are processed along separate routes (see also^5,6^). Later studies have challenged this view (e.g.^7–9^), and some researchers have argued that how sex and identity of faces are processed may depend upon the level of visual analysis^10^. In the present study, by computationally manipulating the sex and identity of faces and training computational models for face categorization, we aimed to further examine how sex and identity of faces are processed and whether artificial and biological visual systems categorize faces in a similar manner.

The interaction between sex and identity processing has been investigated with many tasks. Here the sex of a face refers to the sex category of the face, based on its physical appearance, and we define the identity of a face as its unique facial features that allow people to distinguish that very face from all other faces. Previous studies often approximate the processing of face identity with the processing of face familiarity. For example, Rossion^8^ morphed newly learned faces with same-sex unfamiliar faces to create faces of different levels of perceptual familiarity and found that sex-classification was faster for perceptually familiar faces than for unfamiliar faces. He then proposed that face familiarity is processed before the completion of sex categorization, arguing against the view that determining the sex of a faces occurs first or faster than processing their identity (see also^11^). Goshen-Gottstein and Ganel attempted to tackle the same question using both a priming and a Garner’s task^7,12^. They found that priming speeds up sex-classification, whilst varying face familiarity slows down sex-classification, indicating that identity and sex of faces are not processed completely separately. Zhao and Hayward^9^ adopted a search task with target and distractors either sharing the same sex or not. They found that people were slower and less accurate when target and distractors are of the same sex than when not (see also^13^). They proposed that invariant aspects of faces, like sex, race, and identity, are processed in an integrative manner. That is, processing one such aspect involves automatic and compulsory processing of other invariant aspects of facial information.

Computational studies of face perception also showed an interaction between sex and identity processing. For instance, computational models that are trained to identify faces are not only able to identify faces but also can categorize faces by sex or race without further training^10,14^. When asking face recognition models to decide if two faces display the same person or not, the models can take race-related facial information into consideration^15^. Similarly, “neurons” in a neural network model of a primate visual system can learn to respond exclusively to male or female faces, without being trained to identify faces or to categorize the sex of faces^16^, see also^17^. These studies suggest that characteristics like sex and race can be learned spontaneously through learning or even more simply by viewing faces of different identities, sex and races.

Although all afore-mentioned studies are consistent with an interdependency of identity and sex processing, the nature of such an interaction remains unsettled (cf.^4,18^). The interaction could arise from the crosstalk between separate processing of identity and sex. For instance, one process may occur before, and therefore exert influence on the other^8,13^. Alternatively, these two facial properties might be extracted via the same visual analysis route or rely on partially overlapping facial information, with sex acting as one of many dimensions defining face identity^10,14,16,17^. Previous research often takes an indirect approach to address this question. For behavioral studies, the interaction between identity and sex processing is often demonstrated by introducing task-irrelevant faces or facial properties (e.g., distractors in a search task; variation of sex or familiarity in the Garner task^7,9,12^). A stronger demonstration of such an interaction would be to show how processing of sex and identity in the same face affects each other. In other words, more solid evidence would be obtained through investigating whether the classification of the sex (or identity) of a face is directly impacted when the identity (or sex) of the same face changes. For computational research, although several studies indicate that the visual system may spontaneously learn sex categories while differentiating face identities, these models are not often accompanied by and compared with human performance. Moreover, while familiarity with faces plays an important role in human sex categorization^12,19^, few computational models of face perception have attempted to characterize differences between familiar and unfamiliar face processing. Hence, how these computational models capture the mechanisms underlying identity and sex classification in humans remains elusive.

In the present study, we investigated whether sex and identity of faces are processed integratively in a sex categorization task. Although the influence of identity processing (in terms of familiarity analysis) on sex categorization has been examined by many previous studies (e.g.^5,7,8,11,12^), how sex change may affect the perception of the sex of familiar faces remains to be elucidated. Even less is known about how changes in identity strength (i.e., how much identity specific information is preserved) may alter our perception of the sex of the faces. Here, we examined how sex categorization performance is affected when the sex of faces is computationally swapped (Experiment 1) and when the identity strength of faces is manipulated (Experiment 2). The use of those modified faces in a sex categorization task directly tests the hypothesis that sex information is an integral dimension of identity information. Such a hypothesis is derived from recent computational studies that has not yet been tested with human performance (e.g.^10,14,16,17^). If this hypothesis holds, manipulating either identity or sex of faces should affect both identification and sex classification. Our previous study provides some support for one side of the hypothesis, whereby varying the identity, sex, or race of faces at a fine-grained level affects identity analysis (i.e., ability to identify personally familiar faces^20^). We found that people do not simply encode a familiar face as belonging to dichotomous male/female or Asian/White categories; instead, these dimensions are coded along a continuum (i.e., how much feminine/masculine or Asian/White a face is). Moreover, faces are represented less precisely along the sex or race continuum and often their real values along the continuum are augmented in memory. For example, familiar female/White faces are represented as more feminine/White than they actually are. The current study tested the other side of the hypothesis, that is, varying the identity or sex of faces would also affect the perception of the sex of said faces. In contrast to extensive research on what affects identity analysis in the literature, fewer studies have attempted to characterize what modulates the perception of the sex of faces.

We also investigated how familiarity with faces may modify the effects of identity and sex manipulations on sex categorization. Familiarity with faces here refers to visual knowledge about faces, which could be obtained via passive viewing experience (i.e., visual or recognition familiarity) or real-life personal interaction (i.e., personal familiarity). Previous studies have shown that visual familiarity enhances the perception of various aspects of facial attributes (e.g.^7,11,19^, but see^4–6^). Here we focused on testing how personal familiarity with faces might influence sex categorization when the sex or identity strength of those faces were modified. To this end, we tested two groups of participants (control vs department group) with two sets of faces (unfamiliar vs colleague face set). Participants in the department group were personally familiar with the faces in the colleague face set, whereas neither group was familiar with the unfamiliar face set. The effect of familiarity can be assessed by comparing both groups’ performance on the colleague face set, or by contrasting the department group’s performance on both sets of faces. Moreover, by investigating how familiarity modulates sex classification, we can also differentiate between the influence of familiarity processing from the influence of identity analysis, which are often used interchangeably in previous literature. We manipulated both the strength of face identity (i.e., level of idiosyncratic information) and participants’ familiarity with these faces (i.e., with or without visual knowledge) in Experiment 2. The former allowed us to examine how variation in sex categorization directly connects to variation in identity strength, whereas the latter enabled us to test whether familiarity with faces facilitates sex classification (i.e., an indirect test of the interaction between identity analysis and sex classification^6–8^).

Finally, we investigated whether our manipulation of the sex and identity of faces had a similar effect on the response of humans and computational models. Many computational models have been developed to classify the sex of faces (e.g.^21,22^). While these models sometimes reach or surpass human performance on categorizing the sex of faces, whether they capture the characteristics of human perception of the sex of faces remains to be elucidated. To address this question, we trained three computational models to categorize the sex of faces based on three deep neural network models (AlexNet^23^; ResNet50^24^, and Inception-ResNet-V2^25^). While the underlying architecture differed between these neural network models (8 layers for AlexNet; 50 layers for ResNet50, and 164 layers for Inception-ResNet-V2), they are all highly accurate in image-based object classification and are frequently used when comparing visual object recognition between humans and computational model (e.g.^26^). By comparing model-based responses in the sex categorization task with our participants’ performance, we can infer what is shared and what differs between face categorization in biological and artificial face processing systems.

## Experiment 1

In this experiment, we tested how changes along the sex dimension in faces affect sex categorization, how familiarity with faces influences sex categorization performance, and whether our manipulation has a similar effect on human performance and on responses of computational models trained for sex classification. The experiment involved two groups of participants and two sets of faces. One set of faces consisted of 3D scans of our colleagues’ faces (the *colleague face set*) and the other set was comprised of 3D scans of volunteers’ faces (the *unfamiliar face set*). One group of participants consisted of colleagues within our department (the *department group*); they were personally familiar with the faces in the colleague face set and did not know any of the faces in the unfamiliar face set. Another group of participants were volunteers unfamiliar with both sets of faces (the *control group*). All participants performed a sex categorization task on both sets of faces. All test faces were shown in two versions: an original version and a sex-changed version. For the latter, the faces were computationally manipulated to give the appearance of the opposite sex without changing their identity (see “Methods” section below). If sex and identity are fully separable dimensions of a face, both groups of participants should display comparable sex categorization performance across face sets and face versions. In contrast, if sex is encoded integrally with face identity, they should perform similarly with the *unfamiliar* face set but differently regarding the *colleague* face set. Familiarity with faces should enhance sex categorization performance on the original version of face (e.g.^8^) but may weaken performance on the sex changed version due to the incongruency between perceived and memorized sex categories. We measured participants’ performance using their categorization accuracy and response time for the sex categorization task. For the model-based sex-categorization, performance was measured as categorization accuracy.

### Methods

#### Participants

We tested two groups of participants. The *department group* consisted of 25 members of our department (all were White Europeans; 6 females and 19 males; mean age: 34 years old, between 26 and 42 years old). They were personally familiar with the people whose faces were used in the *colleague face set* and were offered a choice of sweets to thank them for their voluntary participation. Data from one participant who did not follow the instructions was discarded. The *control group* consisted of 23 participants recruited from our participants database (all were White Europeans; 13 females and 10 males; mean age: 27 years old, between 19 and 61 years old). They were paid for their participation, and they all gave informed consent to participate in the study. For our main 2 by 2 repeated measure ANOVAs, a priori power analysis using G*Power 3.1 indicates that 46 participants in total are required to test the main effect face manipulation and its interaction with participants groups with a statistical power (1 − *β*) of 0.90 and a medium effect size (*f* = 0.25) at *α* = 0.05. The procedures were approved by the Ethical Review Board of the Max Planck Society. All methods were performed in accordance with the relevant guidelines and regulations. Both groups were naïve to the purpose of the experiment.

#### Stimuli

The face stimuli were organized in two sets. The *colleague face set* was derived from 3D face scans of 15 colleagues (8 females and 7 males, all were White faces, mean age at face scanning: 35 years old, between 26 and 43 years old). The *unfamiliar face set* was derived from 3D face scans of 13 people unknown to our participants (11 females and 2 males, all were White faces, mean age at face scanning: 27 years old, between 19 and 38 years old). We created two images for each face: an original version was created by using the unaltered scan whereas the sex-changed version was created by computationally changing the appearance of the face scan to the opposite sex. Because of the sex manipulation, there was an equal number of male-looking and female-looking faces in both face sets. All images were shown in full color, rotated 20 degrees to their left, 330 × 330 pixels in size, and subtended a visual angle of approximately 8° by 8°. As our face manipulations (i.e., changing sex or identity strength) were based on 3D face scans, these slightly rotated faces give better view of crucial 3D facial structures (e.g., eyebrow arch and jaw) for sex classification^27^.

**Figure 1:**
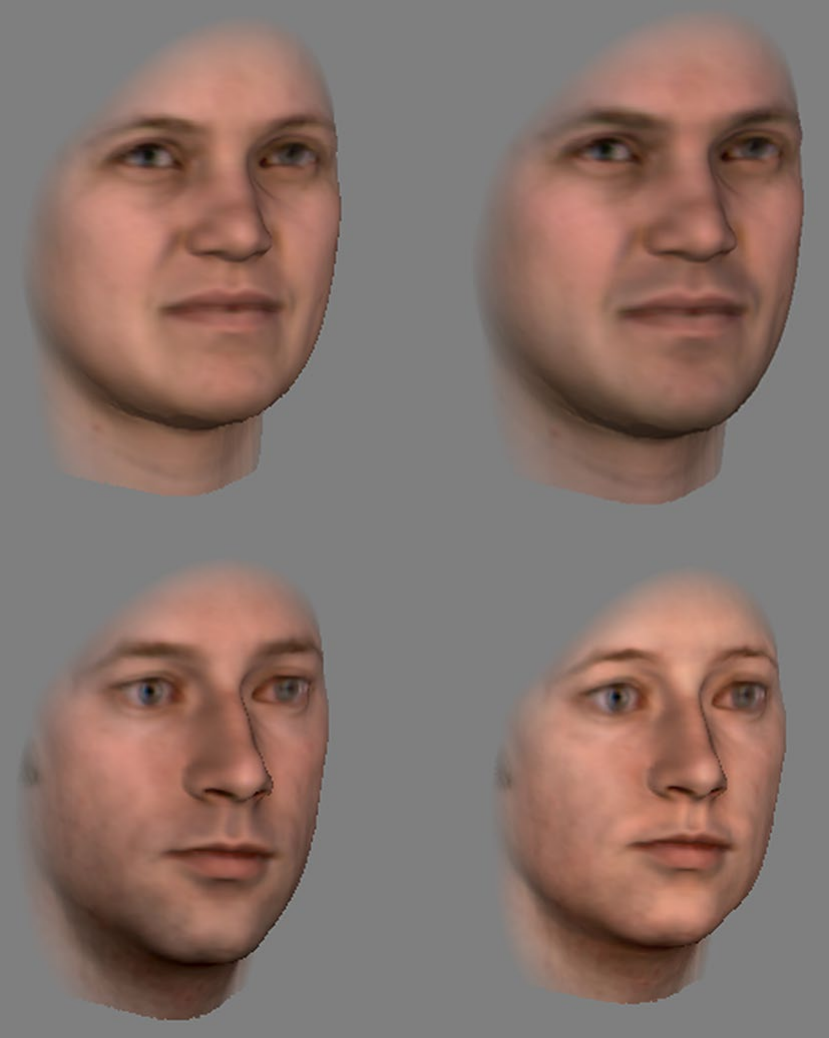
Examples of face stimuli used in Experiment 1. Left column, original faces of a female (top) and a male (bottom). Right column, their modified versions, showing the faces in opposite-sex appearance. Written informed consent was obtained from our colleagues to publish images of their original and manipulated faces in an online open access publication.

To manipulate the sex of faces (e.g., making male faces female-looking and female faces male-looking, Fig. 1), we used the Max Planck Institute face database (http://faces.kyb.tuebingen.mpg.de) and a 3D morphable model^28,29^. All faces in the database are in correspondence in terms of shape and texture (color). To modifying the sex of a face, we first calculated a sex vector defining how a face changes when only the sex of the face is varied. This vector was then applied to the test faces to change specifically their appearance along the sex dimension. The same method has been used to manipulate identity, sex, or race information of faces without changing irrelevant aspects of faces in previous studies^19,20,27,28,30^.

We applied sex change manipulation to male and female faces differently given the well-documented male bias in sex categorization of faces^30–32^. That is, people tend to categorize female faces as male when they are not certain. To address this issue, the sex vector was applied in part (65%) to create male versions of original female faces. Our pilot study showed that such sex-changed faces were classified as male on average in 95% of all trials. In contrast, we applied full vector (100%) to create female versions of original male faces. These female-looking faces were classified as female in 77% of all cases in our pilot study, indicating that the male bias is difficult to eliminate. We did not further enhance the femininity of these faces for the following reasons. First, feminizing the male faces more strongly creates distracting artefacts. Second, these feminized versions elicit comparable sex categorization performance to the original female faces from the same database, which were accurately categorized in 68% of the trials^19^.

#### Procedure

Participants sat in a quiet experimental room and viewed the stimuli on a computer monitor. We used Eprime software (Psychological Software Tools, Pittsburgh, PA) to present face stimuli and collect data. Before the task, the department group saw the original versions of the colleague faces to familiarize them with their appearance as 3D-scanned faces. They saw these faces one after the other in random order twice, with each displayed for 2 s followed by a 1-s blank screen. The control group did not view the faces of the colleague face set before the test so that they were completely unfamiliar with those faces. Neither group had viewed the faces of the unfamiliar set. Before the test phase, both groups performed five practice trials with additional unfamiliar faces. During test, all participants performed the same sex categorization task for all faces. They saw all original and sex-changed images in random order. The 56 test faces (15 familiar faces × 2 versions + 13 unfamiliar faces × 2 versions) were shown one at a time. Each image was shown for 2 s followed by a blank screen which remained until a response was made. Participants were asked to categorize each face by sex. They were instructed to press one button on a keyboard if they perceived the face as female and another button if they perceived the face as male. After a response, a fixation cross followed for 250 ms before the display of the next face. Department participants were told that some test faces might be familiar and others not. All participants were instructed to judge the sex of the face based exclusively on the facial appearance and to respond as quickly and accurately as possible.

#### Model-based sex categorization

To examine whether computational models capture the characteristics of human performance in sex categorization, we trained three classifiers to categorize the sex of faces based on three convolutional neural networks that have been trained to classify images into different object categories, including the AlexNet^23^, ResNet50^24^, and Inception-ResNet-V2^25^. All three models have shown high image categorization accuracy despite their differences in terms of the implementation architecture and the depth of the neural network. Using pretrained versions of these neural networks embedded in the MATLAB Deep Learning Toolbox support package (The MathWorks, Natick, MA), we first extracted the feature representations of training faces using model activations of one fully connected layer (i.e., layer “fc7” for AlexNet; layer “fc1000” for ResNet50, and layer “predictions” for Inception-ResNet-V2). The features extracted from male and female training faces were then used as predictor variables and fitted to a support vector machine (SVM) classifier (implemented using a MATLAB function, fitcecoc). Finally, we used the SVM classifier to predict both the sex category and its corresponding posterior probabilities. For the latter, we transformed the classification scores for each category (i.e., male, female) to category-based posterior probabilities. Therefore, the models not only tell whether a given face is a male or female (i.e., categorization responses) but also estimate how likely a given face is showing a male and female respectively (i.e., probability responses).

The three models were trained using the same 122 faces from the Max Planck face database (64 were White faces, including 32 male and 32 female faces; 58 were Asian faces, including 27 male and 31 female faces). We used both White and Asian face stimuli to enlarge the number of training instances due to the limited number of White face stimuli available in the database. None of these training faces were used in the main study. These face images were re-sized to fit with corresponding input format of the three neural networks. The training images depict faces that are slightly rotated to the left (15°), which was similar to the facial images used in the main studies. To test the performance of the classifier we first split our training stimuli, using 70% of these images to train the models (same number of male and female faces) whilst using the subsequent 30% of images to test it. We conducted this training and test procedure ten times and found that the mean (± SD) categorization accuracy was 95 ± 3%, 96 ± 3% and 85 ± 7% for AlexNet, ResNet50, and Inception-ResNet-V2 respectively. Thus, all classifiers could successfully learn the diagnostic features to differentiate between male and female faces. We then re-trained the classifiers using all training faces and used them to categorize the sex of all test face images in Experiments 1.

### Results

For human performance, we measured both categorization accuracy and response time, whereas for model-based responses, we only obtained response accuracy data. For both accuracy and response time data, we first performed an omnibus mixed 2 × 2 × 2 ANOVA, with face set (colleague set vs unfamiliar set) and face manipulation (original versions vs sex-changed versions) as within-participants factors and participants group (department group vs control group) as between-participants factor. The omnibus ANOVA displayed many interactions between the three factors. To unpack these interactions, we performed separate ANOVAs for categorization performance on each face set, with face manipulation as a within-participants factor and participant group as a between-participants factor. To make meaningful interpretation of the data and make it easy to follow, we focus on reporting the results of the two separate ANOVAs and place the full results of the omnibus ANOVAs in Supplementary Material Section A. For brevity, here we focus on categorization accuracy data obtained from our participants and the responses of three computational models. Participants’ response time data are reported in Supplementary Material Section B for completeness, which showed a generally consistent patterns of response with the accuracy data and showed no speed-accuracy trade-off. For both experiments reported here, we report values of performance in parentheses in the format of *Mean* ± *Standard error of the mean.*

#### Accuracy

**Figure 2:**
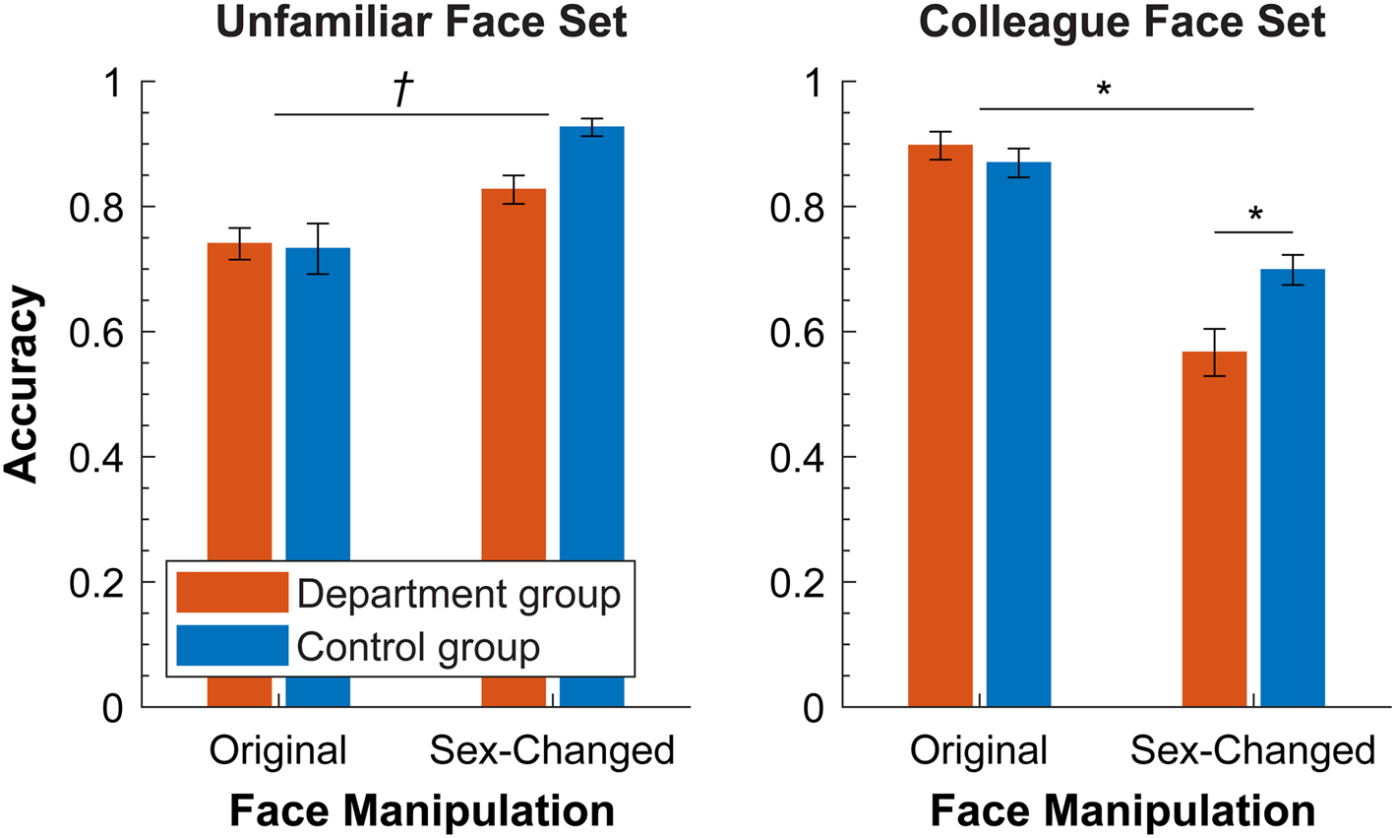
Mean accuracy data in Experiment 1 for each face set. Error bars represent standard errors of the means (SEMs). Long horizontal bars indicate significance of interaction, and short bars indicate significance of follow-up contrast. *p < 0.01; ^†^*p* < 0.10.

Mean accuracy data for each condition and each participants group are shown in Fig. 2. For the *unfamiliar face set,* the ANOVA revealed a main effect of face manipulation, *F*(1,44) = 23.302, *p* < 0.001, *ηp*^2^ = 0.341. Sex-manipulated faces (0.877 ± 0.013) were better categorized than the original faces (0.738 ± 0.024). The control group performed slightly better than the department group (0.830 ± 0.018 versus 0.785 ± 0.018), but the main effect of participant group did not reach significance, *F*(1,45) = 3.229, *p* = 0.079, *ηp*^2^ = 0.067. There was no significant interaction between face sets and participant group, *F*(1,44) = 3.41, *p* = 0.071, *ηp*^2^ = 0.070. These results indicate that altering the sex of faces changed categorization of unfamiliar faces in both groups of participants similarly.

For *the colleague face set*, the ANOVA revealed a main effect of face manipulation, *F*(1,45) = 75.142, *p* < 0.001, *ηp*^2^ = 0.625. Original faces were better categorized than sex-changed ones (0.883 ± 0.016 vs 0.632 ± 0.023). The control group performed slightly better than the department group (0.784 ± 0.019 vs 0.731 ± 0.018); the main effect of the participant group was marginally significant, *F*(1,45) = 4.036, *p* = 0.051, *ηp*^2^ = 0.082. Crucially, there was a significant interaction between participants group and face manipulation, *F*(1,45) = 7.639, *p* = 0.008, *ηp*^2^ = 0.145. Follow-up t-test showed that whereas both groups had equivalent performance on original faces, *t*(45) = 0.847, *p* (uncorrected) = 0.402, Cohen’s *d* = 0.247, department group participants were worse at categorizing the sex-changed faces than the control group, *t*(45) = 2.945, *p* (uncorrected) = 0.005, Cohen’s *d* = 0.859. Therefore, while varying the sex of faces impaired performance of both groups, it had a significantly stronger effect for people who were familiar with the original faces than those who were not.

#### Model responses

**Figure 3:**
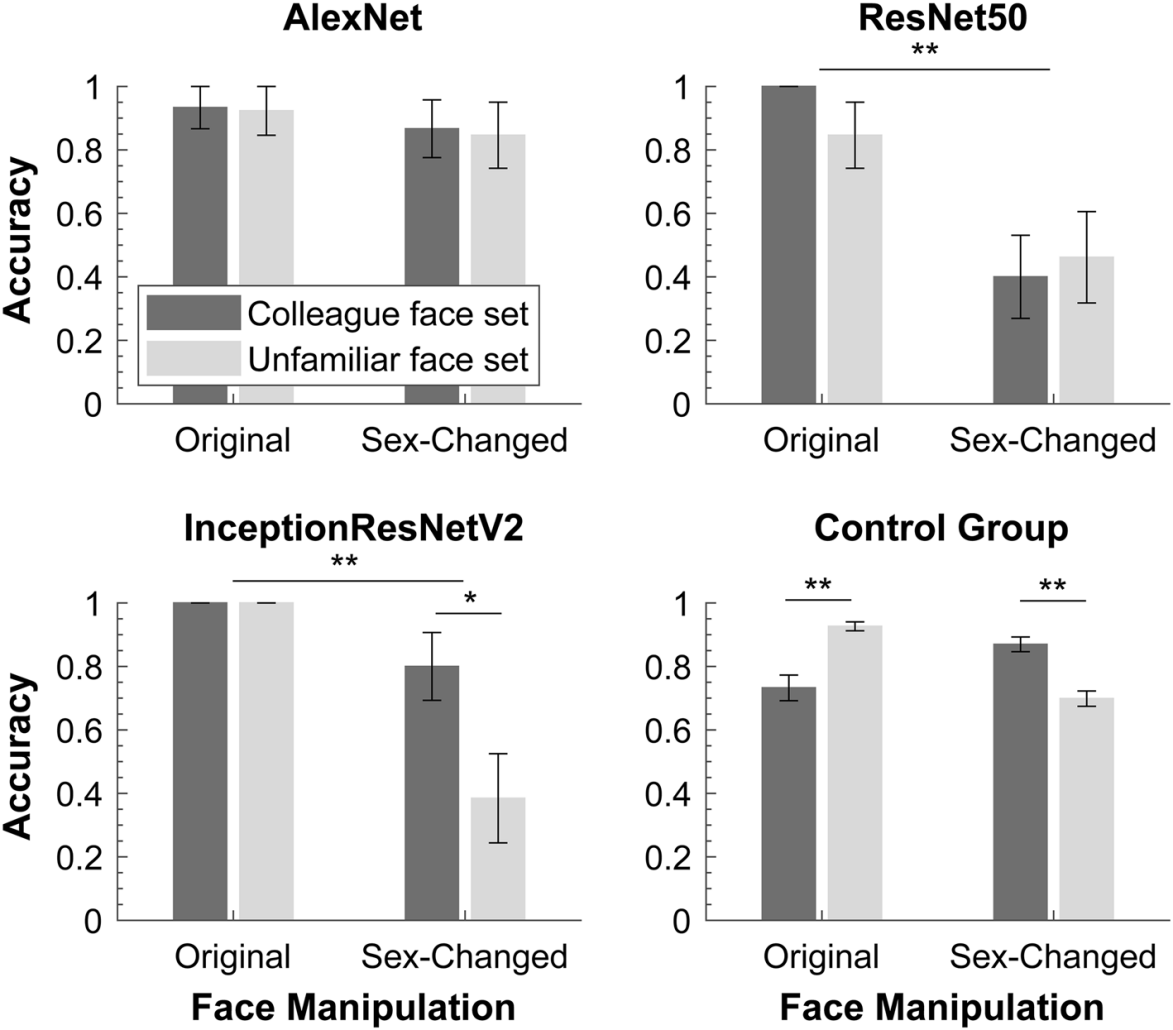
Model-based sex categorization performance in Experiment 1. The results of the control group are replotted here to illustrate the pattern of response observed with humans. Long horizontal bars indicate significance of face manipulation effect, and short bars indicate significance of follow-up contrast. **p < 0.001; *p < 0.05. Error bars represent SEMs. Note that the error bars in some conditions (like those with near perfect performance) were too small to appear clearly here.

Sex categorization responses of the three models are shown in Fig. 3. For all three models, probability responses display patterns of responses similar to their sex categorization accuracy data reported here (see Supplementary Material Section C for detailed results). To examine whether the models responded differently to both face sets and to test whether their responses are modulated by face manipulations, we performed an item-based 2 (face set) × 2 (face manipulation) ANOVAs using model responses to individual faces. We treated face manipulation as a within-face factor as the original and the sex-changed faces were always paired (i.e., derived from the same identity), and we treated face set as a between-face factor as they were independent of each other. This way of analysis also allowed a direct comparison with participants’ performance. Since all test faces were “unfamiliar” to the model, we would expect a similar pattern of model responses to human performance observed with our control group.

As shown in Fig. 3, the three models showed different patterns of responses and none of them was similar to human performance. Similar patterns of responses were also observed when model-based sex categorization was measured using probability responses (see Supplementary Material Section C for detailed results). For AlexNet responses, neither of the two main effects nor their interaction was significant, all *F*s < 1, all *p*s ≥ 0.432, all *ηp*^2^ ≤ 0.024. These results suggest that the model does not categorize the two face sets differently (colleague face set: 0.900 ± 0.055, unfamiliar face set: 0.885 ± 0.059), and more importantly, that reversing the sex of faces does not significantly affect model-based sex categorization (Original faces: 0.928 ± 0.051, sex-changed faces: 0.856 ± 0.069).

For responses of the ResNet50, we only found a significant main effect of face manipulation, *F*(1,26) = 16.441, *p* < 0.001; *ηp*^2^ = 0.387. Sex categorization was better for original faces (0.923 ± 0.048) than for sex-changed faces (0.431 ± 0.097). The main effect of face set and its interaction with face manipulation were not significant, both *F*s < 1, *p*s ≥ 0.383, both *ηp*^2^ ≤ 0.029. Thus, changing the sex of faces impacted sex categorization of the ResNet50 model similarly for both face sets.

For responses of the Inception-ResNet-V2, both the main effect of face manipulation, *F*(1,26) = 21.981, *p* < 0.001; *ηp*^2^ = 0.458, and face set, *F*(1,26) = 5.704, *p* = 0.024; *ηp*^2^ = 0.180, were significant. Their interaction was also significant, *F*(1,26) = 5.704, *p* = 0.024; *ηp*^2^ = 0.180. Follow-up t-tests revealed that, for the original faces, there was no differences between the two face sets (both were 100% accurate), whereas for the sex-changed faces, categorization was more accurate for the colleague face set (0.800 ± 0.119) than for the unfamiliar face set (0.385 ± 0.127), *t*(26) = 2.388, *p* (uncorrected) = 0.024, Cohen’s *d* = 0.905. Therefore, changing the sex of the faces impacted sex categorization of the Inception-ResNet model, but more so for the unfamiliar face set than the colleague face set.

### Discussion

All results of Experiment 1 are summarized in Table 1. These results demonstrate that manipulating the sex of faces affects sex categorization of familiar and unfamiliar faces differently. The effect of face familiarity was tested by contrasting performance of both groups on the colleague face set (those faces were personally familiar only to the department group). While our participants in both groups were better at categorizing original than sex-changed colleague faces, sex manipulation impaired categorization performance of the department group significantly more than the controls. This pattern of response was also observed in response time data (Supplementary Material Section B). Thus, changing face in terms of their sex induced more categorization errors to colleague faces when they were familiar than when they were unfamiliar (Figs. 2 and 3). These findings point to a negative effect of familiarity on the categorization of sex-changed faces. Participants displayed difficulty at classifying faces in terms of their sex using exclusively their appearance without any influence of identity processing.

**Table 1:** Summary of the main results of Experiment 1.

	Participants’ performance		Model responses		
	Unfamiliar faces	Colleague faces	AlexNet	ResNet	Inception
Face manipulation	O < S	O > S	ns	O > S	O > S
Participants group^1^	ns	ns	ns	ns	Colleague > unfamiliar
Interaction^1^	ns	O: ns S: Control > Department	ns	ns	O: ns S: Colleague > Unfamiliar

ns, not significant; O = original faces; S = sex-changed faces; Colleague = Colleague face set; Unfamiliar = Unfamiliar face set. > better than; < worse than. ^1^For model responses, face set (not participant group) was a factor in the ANOVA.

Two seemingly surprising results are worth mentioning. First, we did not obtain any evidence of a beneficial familiarity effect on sex categorization (cf.^8,11^). Participants in the department group, who personally knew the original faces in the colleague set, did not show significantly higher accuracy response to those faces than the control group (Fig. 2 and Supplementary Fig. S1). The lack of a positive familiarity effect might be due to the presence of sex-changed faces of colleagues that caused participants of the department group, but not the control group, to be generally hesitant in their sex categorization task. Second, for the unfamiliar face set, performance was higher for sex-changed version than for the original version. This result is probably driven by the male bias in sex classification. The unfamiliar face set consisted of unbalanced number of male and female faces in (11 female and 2 male faces), which made their sex-changed versions predominantly male. As male faces are generally better categorized than female faces and female faces are often mistaken as male faces^29,32–35^, the original faces (mostly female) in the unfamiliar face set were categorized with lower accuracy than their sex-changed versions (now mostly male-looking). This male bias should not apply to the colleague faces set, which contained similar numbers of male and female original faces (8 females and 7 males).

Note that our study was limited by the availability of face scans (i.e., stimuli) and colleagues (i.e., participants), which resulted in unbalanced numbers of male and female faces in our unfamiliar face set. It led to the observed higher categorization accuracy for the sex-changed faces in the unfamiliar set, which, as discussed above, was due to a male bias in sex categorization^32–35^. Nonetheless, this factor cannot account for our main results. Specifically, if sex change did not play any role in sex categorization, then for both face sets we would expect equivalent performance for the original and the sex-changed faces, regardless of the level of performance for the original faces. This is not the case in our study. Similarly, if familiarity with faces did not play any role in the influence of sex change on sex categorization, we would expect no interaction between participants group and sex manipulation, regardless of whether sex change increases or impairs sex categorization performance. This is not the case either. Finally, it may be argued that stimuli-based differences between colleague face set and unfamiliar face set might make sex-changed faces more difficult to categorize for the former than the latter, thereby eliciting impaired categorization performance for the colleague face set. Our model-based responses indicate that it was unlikely as none of the three models exhibit lower categorization accuracy for sex-changed faces of colleague face set than for those of the unfamiliar face sets.

Changing the sex of faces had differing influences on participants’ performance and model-based responses. Our participants showed significant differences between categorizing the original and the sex-changed versions of faces, either due to familiarity with the original faces (i.e., colleague face set) or because of a male bias (i.e., unfamiliar face set). The computational models trained to classify the sex of faces, however, either showed no such response difference (i.e., AlexNet) or showed response difference in the opposite direction (i.e., responses of ResNet50 and Inception-ResNet-V2 to unfamiliar face set). These results indicate that model-based sex categorization may not capture the full characteristics of human sex classification. In contrast to our participants, the AlexNet model seems to use exclusively the visual features it learned for sex-decision and therefore categorized male and female faces without showing a male bias in sex categorization^32–35^. The ResNet50 and Inception-ResNet-V2 models showed almost perfect sex categorization for the original faces, however, these models have difficulty to transfer the learned diagnostic features to classify sex-changed faces, particularly for the unfamiliar face set. Altogether, these results also suggest that our participants’ different performance with original and sex-changed faces cannot be solely attributed to the image-based differences between the two sets of faces.

## Experiment 2

Experiment 2 investigated how varying face identity strength may affect sex categorization of human observers, whether this effect may be modulated by face familiarity, and whether the model trained for sex classification would responds similarly to humans. Thus, we explore the same aspects of sex categorization as in Experiment 1, but with other face manipulations. Previous studies on the interaction between identity and sex processing often used face familiarity to approximate identity processing and seldomly differentiated between them (e.g.^7–9,12^). In this experiment, we manipulated both the familiarity (personally known, unknown) and identity (caricatures, original, anti-caricatures) of the faces, so that we could examine the roles of both familiarity processing (at a coarse level) and identity analysis (at a fine-grained level) in sex categorization. Same as Experiment 1, we manipulated face familiarity by testing both a department group and a control group of participants with two sets of faces (colleague vs unfamiliar face set). To manipulate face identity, we created three versions of faces that either reduce (i.e., anti-caricatures), preserve (i.e., originals), or enhance (i.e., caricatures) the strength of identity information. Therefore, from an anti-caricature to the original face and further to a caricature, each test face gradually moves away from the average face (i.e., face prototype) and its identity strength gradually increases. If sex and identity are integral aspects of face recognition, altering the strength of face identity (by enhancing or reducing idiosyncratic facial information) should affect sex categorization of these faces.

How may sex classification change with increasing identity strength? One possibility is that enhancing identity strength augments all identity-related information, including the sex of a face. In other words, caricatures may not only make a long nose even longer, but may also make a male face looks more masculine, thereby improving sex categorization. Alternatively, enhancing identity strength may weaken facial information defining the sex of a face, since it pushes the face further away from the prototypical male/female average face. That is, in comparison to the original faces, caricatures may make faces less prototypical in terms of sex, thereby impairing sex categorization. It might also be possible that facial information defining identity is independent of that defining the sex of a face. If this the case, increasing identity strength should have no effect on sex categorization.

### Methods

#### Participants

Two groups participated in this experiment. The *department group* consisted of 20 colleagues of our department (9 females and 11 males, mean age: 34 years old, between 22 and 61 years old; all were white Europeans except for one Asian colleague); they were offered a choice of sweets to thank them for their participation. Eight participants (2 female and 6 male) had participated in Experiment 1. The *control group* consisted of 20 students recruited from the University of East Anglia (18 females and 2 males; mean age: 20 years old, between 18 and 23 years old; all were white Europeans). They obtained course credit for their participation, and all gave informed consent to participate in the study. Both groups of participants were naïve to the purpose of the experiment. For a 2 by 3 repeated measure ANOVAs, a priori power analysis using G*Power 3.1 indicates that 36 participants in total are required to reach statistical power (1 − *β*) of 0.90 with medium effect size (*f* = 0.25) at *α* = 0.05.

#### Stimuli

**Figure 4:**
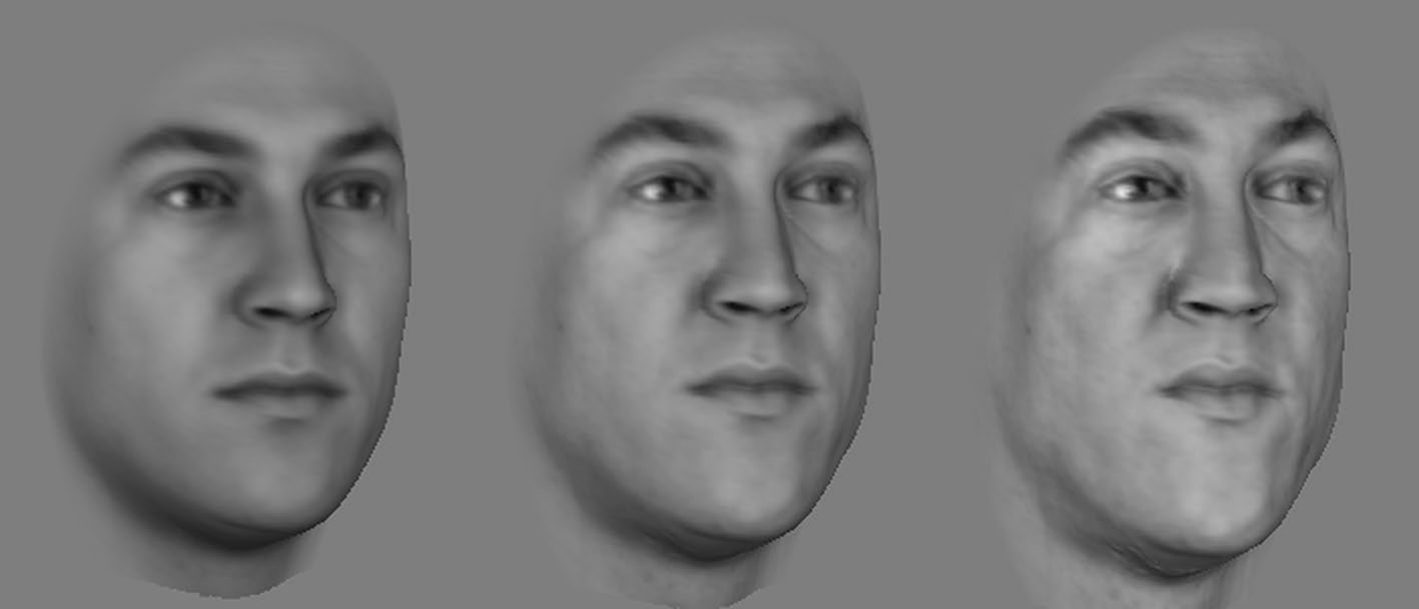
Examples of face stimuli used in Experiment 2. From left to right, anti-caricature (60% identity strength), original (100% identity strength), and caricature (140% identity strength) of a male face. Informed consent was obtained from our colleague to publish images of his original and manipulated faces in an online open access publication.

There were two sets of face stimuli. The *colleague face set* was created from 3D-scans of 14 faces of members of our department (8 females and 6 males; all were White faces, mean age at face scanning: 36 years old, between 26 and 43 years old). The *unfamiliar face set* was created using 3D-scans of 13 faces unknown to our participants (6 females and 7 males; all were White faces, mean age at face scanning: 27 years old, between 19 and 38 years old). For each face, we created three pictures (Fig. 4): its original version, its caricatured version (i.e., showing enhanced idiosyncratic information), and its anti-caricatured version (i.e., with reduced idiosyncratic information). These faces were shown in grey to reduce the potential impact of color artefacts produced by the caricaturization method. The facial images measured 330 × 330 pixels in size and subtended a visual angle of approximately 8° by 8°.

Caricatures and anti-caricatures were created using the same methods as in previous studies (for more details see^20,36,37^). Specifically, we 3D-morphed the original face either toward the average face (i.e., creating an anti-caricature) or away from the average face (i.e., creating a caricature). To do so, we first calculated the shape and texture differences between an original face and the average face. These differences were then either enhanced to create caricatures (e.g., a long nose would become even longer) or reduced to create anti-caricatures (e.g., a large nose would be reduced toward the average nose size) in terms of a face space framework^38^. We created a male and a female average face and generated caricatures and anti-caricatures using a sex-match average face to avoid manipulating faces in terms of their sex. We reduced the shape and texture difference between originals (100% identity strength) and average face (0% identity strength) to create anti-caricatures with 60% identity strength. Conversely, we increased the differences to create caricatures with 140% identity strength. Thus, while anti-caricatures and caricatures were at the same computational distances from the original faces, one was closer to the average face and the other was further away from the average face than the original face. In other words, the strength of face identity was linearly increased from anti-caricatures (60%) to original faces (100%) and further increased to caricatures (140%).

#### Procedure

All participants performed the same sex categorization task for both sets of faces. Before the task, the department group saw the original versions of the colleague faces to familiarize them with their appearance as 3D-scanned faces. They saw these faces one after the other in random order twice. The control group had no such familiarization procedure. Before the test phase, all participants performed 12 training trials with unfamiliar faces that were not used in the test. The 81 test faces (14 familiar faces × 3 versions + 13 unfamiliar faces × 3 versions) were shown once. All other aspects of the procedure were the same as described in Experiment 1.

#### Model-based sex categorization

To examine whether the characteristics of participants’ sex categorization can be captured by computational models, we also obtained model-based responses to all faces used in Experiment 2. The computational models, training stimuli and the methods were the same as those used in Experiment 1.

### Results

As in Experiment 1, we first performed a mixed 2 × 3 × 2 ANOVA on the accuracy data with face set (colleague set vs unfamiliar set) and face manipulation (anti-caricature, original, caricature) as within-participants factors and participants group (department vs control group) as between-participants factor. This omnibus ANOVA displayed many interactions between the three factors. To unpack these interactions, we performed separate ANOVAs for categorization performance on each face set, with face manipulation as a within-participants factor and participant group as a between-participants factor. Here we focus on the results of these two separate ANOVAs and place the full results of the omnibus ANOVAs in the Supplementary Material Section A. Similar to Experiment 1, we reported response time data and their analyses in Supplementary Material Section B for completeness.

#### Accuracy

**Figure 5:**
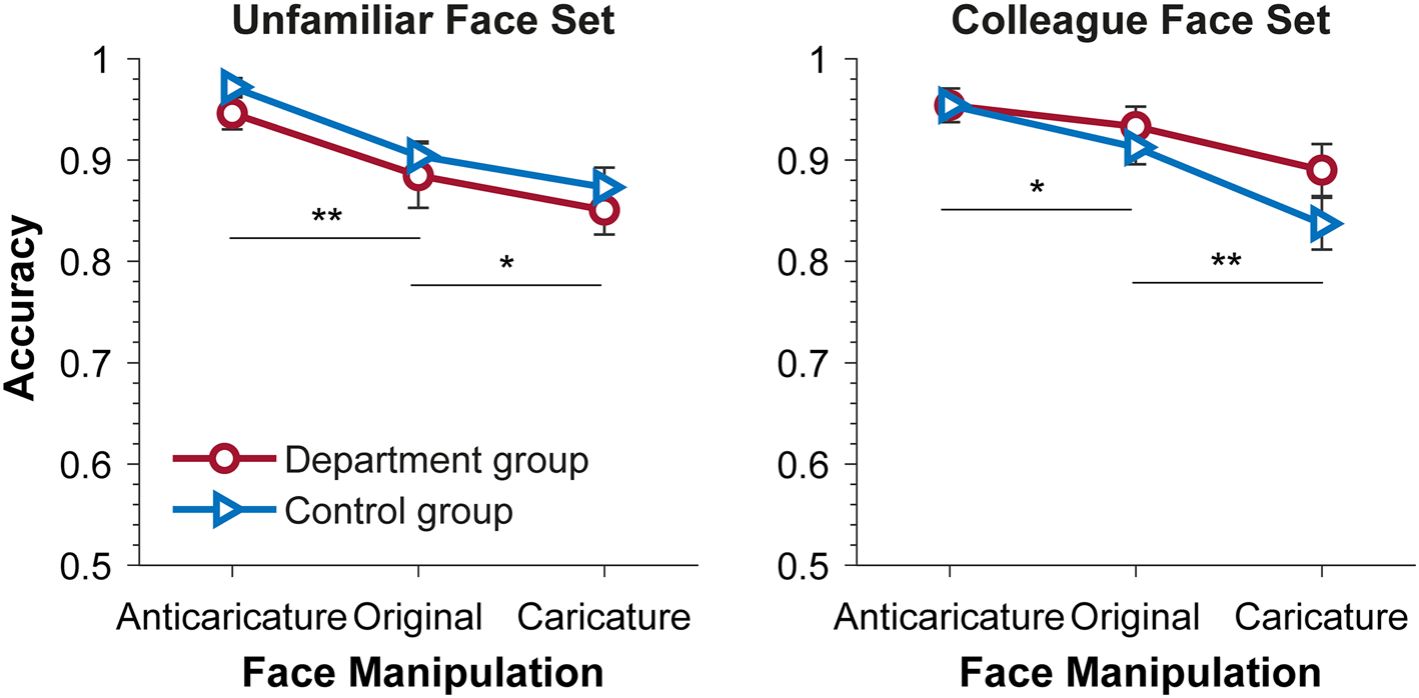
Mean accuracy data in Experiment 2 shown for each face set. Error bars represent SEMs. Horizontal bars indicate significance of difference between face manipulation conditions. **p < 0.005; *p < 0.05.

Figure 5 shows the results of response accuracy in Experiment 2. For the *unfamiliar face set,* a 3 (face manipulation: anti-caricature, original, caricature) by 2 (group: unfamiliar vs department group) ANOVA revealed a main effect of face manipulation, *F*(2,76) = 17.839, *p* < 0.001, *ηp*^2^ = 0.319. Within-participants contrasts showed that sex categorization performance was higher for anti-caricatures (0.959 ± 0.009) than for original faces (0.894 ± 0.017), *F*(1,38) = 14.283, *p* < 0.001, *ηp*^2^ = 0.273, which was then higher than that for caricature faces (0.862 ± 0.016), *F*(1,38) = 4.172, *p* = 0.048, *ηp*^2^ = 0.099. This ordered pattern of response was similar for both groups of participants; neither the main effect of participants group,* F*(1,38) = 1.093, *p* = 0.302, *ηp*^2^ = 0.028, nor its interaction with face manipulation, *F*(2,76) = 0.019, *p* = 0.981, *ηp*^2^ < 0.001, was significant. These results indicate that manipulating identity strength of faces affects their sex categorization; increasing identity strength leads to reduced performance on sex classification.

For *the colleague face set,* the same ANOVA revealed a main effect of face manipulation, *F*(2,76) = 16.785, *p* < 0.001, *ηp*^2^ = 0.306. Within-participants contrasts showed that sex categorization performance was higher for anti-caricatures (0.954 ± 0.012) than for original faces (0.923 ± 0.013), *F*(1,38) = 4.882, *p* = 0.033, *ηp*^2^ = 0.114, which was subsequently higher than that for caricature faces (0.864 ± 0.018), *F*(1,38) = 10.795, *p* = 0.002, *ηp*^2^ = 0.221. This ordered pattern of response was similar for both groups of participants; neither the main effect of participants group,* F*(1,38) = 1.168, *p* = 0.287, *ηp*^2^ = 0.030, nor its interaction with face manipulation, *F*(2,76) = 1.418, *p* = 0.248, *ηp*^2^ = 0.036, was significant. Therefore, while sex categorization is affected by changing face identity strength, it is not influenced by participants familiarity with those faces.

#### Model responses

**Figure 6:**
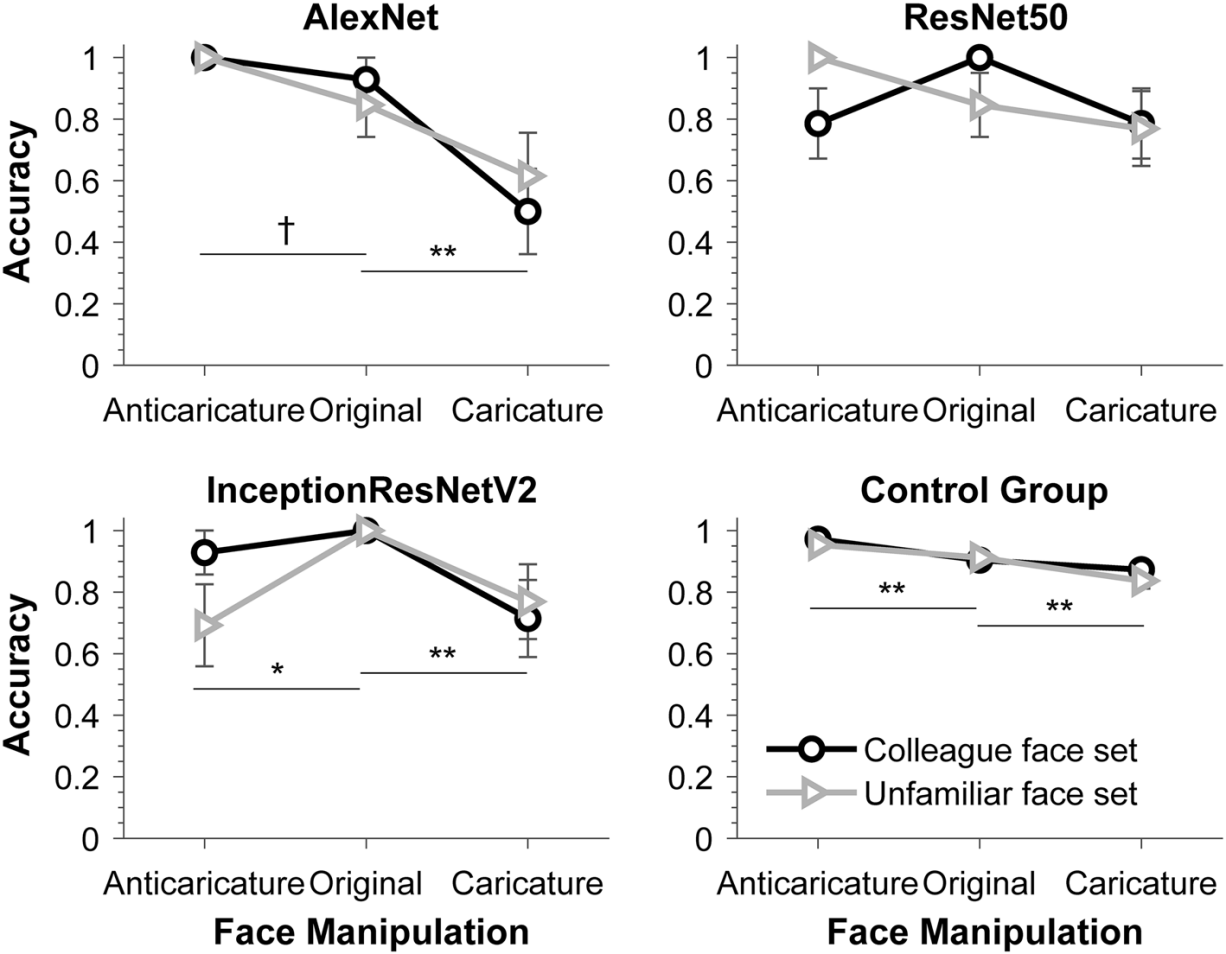
Sex classification performance of the computational models in Experiment 2. The results of the control group are replotted here to illustrate the pattern of response observed with humans. Horizontal bars indicate significance of difference following a main effect of face manipulation. **p < 0.01; *p < 0.05; ^†^*p* < 0.10. Error bars represent SEMs. Note that the error bars in some conditions (like those with near perfect performance) were too small to appear clearly here.

Model-based sex categorization performance for each face set and face condition are shown in Fig. 6. Similar to Experiment 1, to examine whether the model responded differently to the two sets of faces and whether it is affected by the manipulation of face identity, we performed an item-based 2 (face set) × 3 (face manipulation: anti-caricature vs original vs caricature) ANOVAs using model responses to individual faces. As in Experiment 1, we treated face manipulation as a within-face factor and face set as between-face factor.

As shown in Fig. 6, the three models responded differently to the sex categorization task, and only AlexNet showed a pattern of response similar to that observed with our control group. For all models, probability responses display patterns similar to their sex categorization accuracy data (see Supplementary Material Section C for detailed results). For AlexNet responses, the ANOVAs revealed a significant main effect of face manipulation, *F*(2,50) = 14.309, *p* < 0.001, *ηp*^2^ = 0.364. Follow-up contrasts showed that model-based sex categorization performance was numerically higher, though not significantly, for anti-caricatures (1.000 ± 0.000) than for original faces (0.887 ± 0.062), *F*(1,25) = 3.263, *p* = 0.083, *ηp*^2^ = 0.115. Performance to the latter was significantly higher than for caricature faces (0.558 ± 0.099), *F*(1,25) = 12.771, *p* < 0.001, *ηp*^2^ = 0.338. This decreasing pattern of responses appears for both sets of faces. Neither the main effect of face set, *F*(1,25) = 0.014, *p* = 0.905, *ηp*^2^ = 0.001, nor the interaction between face set and face manipulation, *F*(1,25) = 0.668, *p* = 0.517, *ηp*^2^ = 0.026, was significant. This pattern of responses mirrors our participant performance, though increasing identity strength appears to have a stronger effect on model responses than on human performance.

For responses of the ResNet50, neither the two main effects nor their interaction was significant, all *F*s ≤ 2.238, *p*s ≥ 0.117, all *ηp*^2^ ≤ 0.082. Thus, manipulation of face identity strength does not affect sex categorization of the ResNet model, and it shows no difference between the two sets of faces.

For responses of the Inception-ResNet-V2, there was only a significant main effect of face manipulation, *F*(2,50) = 3.988, *p* = 0.025; *ηp*^2^ = 0.138. The main effect of face set and its interaction with the face manipulation was not significant, both *F*s ≤ 1.334, *p*s ≥ 0.273, both *ηp*^2^ ≤ 0.051. Follow-up contrasts showed that sex categorization performance was higher for original faces (1.000 ± 0.000) than for both anti-caricatures (0.810 ± 0.074), *F*(1,25) = 6.550, *p* = 0.017, *ηp*^2^ = 0.208, and caricature faces (0.742 ± 0.088), *F*(1,25) = 8.704, *p* = 0.007, *ηp*^2^ = 0.258. No difference was found between the latter two conditions, *F* < 1. Therefore, in comparison to original faces, enhancing and reducing identity strength both impaired sex categorization performance of the Inception-ResNet-V2 model.

### Discussion

Experiment 2 showed three main results (Table 2). First, manipulating identity by enhancing or reducing identity strength affected sex categorization of both familiar and unfamiliar faces. This result provides a direct and strong evidence for the hypothesis that sex and identity are integrative dimensions of faces. Second, sex categorization performance decreased with increasing identity strength, regardless of participants familiarity with the faces. This finding suggests that exaggerating identity-specific information in faces impairs extraction of sex-relevant information (thereby impairing sex categorization), and that weakening identity strength facilitates extraction of sex information (hence improving sex categorization). Third, while AlexNet showed a decreasing sex categorization performance with increasing identity strength, which is similar to the pattern of human performance, the other two models (RestNet50 and Inception-ResNet-V2) did not capture this characteristic of human sex categorization.

**Table 2:** Summary of the main results of Experiment 2.

	Participants’ performance		Model responses		
	Unfamiliar faces	Colleague faces	AlexNet	ResNet	Inception
Face manipulation	A > O > C	A > O > C	A = O > C	ns	A < O > C
Participants Group^1^	ns	ns	ns	ns	ns
Interaction^1^	ns	ns	ns	ns	ns

ns, not significant; A, anti-caricature faces; O, original faces; C, caricature faces; >, better than; =, equivalent to; <, worse than. ^1^For model responses, face set (not participants group) was analyzed as a factor in the ANOVA.

Although both groups also had different numbers of male and female participants, such a difference is unlikely to underlie our main results. Both groups show equivalent categorization performance for the original faces and the same pattern of response for the unfamiliar face set. Moreover, if the change of identity strength would not affect sex categorization, we would expect equivalent performance across originals, caricatures and anti-caricatures for both face sets, which was not the case. Unlike in Experiment 1, here we found no evidence of a familiarity effect. Although the department group showed numerically higher categorization accuracy for the colleague faces than for the unfamiliar faces, it did not reach statistical significance.

## General discussion

We had three goals in the present study. First, we investigated how sex categorization by human observers is affected when sex or identity information of the test faces is manipulated. Second, we asked whether a computational model accomplishing the same sex categorization with the same stimuli displays similar effects of those manipulations on its performance. Third, we tested whether participants respond similarly when familiar and unfamiliar faces were altered. Our results reveal that (1) manipulating sex or identity of faces affects their sex categorization; (2) computational models trained to categorize the sex of faces showed generally different patterns of responses from those observed with human participants; and (3) familiarity with original (unmodified) faces impaired participants’ sex categorization for faces modified along the sex dimension, but not for faces with modified identity strength. These findings not only provide further evidence for integrative processing of sex and identity in face perception but also offer insights into similarities and differences between human and model-based face categorization.

When faces were computationally modified into the opposite sex (e.g., a male colleague’s face appears as a female-looking face, Experiment 1), participants who knew personally the original faces showed worse sex categorization performance than those who were unfamiliar with those faces. No such difference was observed for the original faces. Note that participants had been explicitly instructed to give a response based on the appearance of the faces regardless of their potential familiar look. This detrimental effect of familiarity (i.e., identification of familiar identity despite changes in facial appearance) on sex categorization suggests that sex and identity information in a face are interdependent; it is difficult to categorize the sex of a face without influences from processing of face identity. This result is consistent with previous findings^7–12,14^. Furthermore, by manipulating both the sex and familiarity of faces in a sex categorization task, our study provides a more complete test of how sex and identity interact in face perception than previous research that manipulated sex or identity information alone (cf.^8,9^).

We found impaired sex categorization for familiar, sex-changed faces; this is reminiscent of the Stroop effect^39^. In a typical Stroop experiment, performance on naming the font colors of words is impaired (i.e., longer response time and/or more errors) when the meaning and the color of the words are incongruent (e.g., “red” printed in green font color) than when they are congruent (e.g., “red” printed in red color). In our study, performance on the sex categorization task drops when the apparent and original sex of the familiar faces are mismatched (i.e., in sex-changed faces) than when they match with each other. Just as people have difficulty to focus exclusively on the “face value” of the color of words in a classical Stroop effect, our participants are unable to use solely the visual featural information for categorizing the sex of familiar faces. Contrasting to a typical Stroop paradigm, the irrelevant information to be ignored in our task was the original sex of familiar faces, which is not shown but integrated with the perceived identity. That is, our participants could not easily treat sex and identity information in faces separately.

Both human and model classification performance in Experiment 2 provides further evidence that sex and identity are perceptually interdependent. Several computational studies have proposed that categorical information about faces, like sex and race, might be a byproduct of identity learning as computational models trained to differentiate individual faces can spontaneously classify the sex or race of faces^10,14,16,17^. If, as these computational models suggest, sex or race information is coded inherently with face identity, then directly manipulating face identity should affect the perception of the sex of that face. Our Experiment 2 supports the idea that sex and identity of faces are integral dimensions in face perception. When identity-specific facial information is enhanced (i.e., using caricatures), our participants’ sex categorization performance declines. In contrast, when we reduce idiosyncratic facial information defining face identity (i.e., using anti-caricatures), participants’ performance increases. More importantly, when we trained deep neural network models to classify the sex of faces, the AlexNet model exhibits a similar pattern of responses as observed with our participants. In contrast, RestNet and Inception-ResNet models showed different patterns of results from human performance. Varying identity strength showed no significant influence on responses of the ResNet model, whereas the Inception-ResNet Model showed worse categorization of anti-caricatures than the original faces—a reversed pattern to human performance. Despite different patterns of responses across human and model-based sex categorization, these results suggest that identity strength could affect sex classification in both human and model-based face-processing systems.

Participants’ performance in Experiment 2 (Fig. 5) also hints at how identity and sex information may be integrated in face representation—augmenting identity information seems to weaken sex information in faces. Note that we used same-sex average faces to create (anti-) caricatures to ensure that only idiosyncratic information was altered, not sex information. Anti-caricatures of male (female) faces are more similar to the average male (female) face; their characteristic idiosyncratic features (for example, an asymmetric face) are reduced. In contrast, our caricatures display amplified idiosyncratic characteristics of the original faces, for example a longish face will become more elongated, whereas their masculinity or femininity is not modified. The binding of identity and sex information observed here concurs with our recent findings^20^. When participants were asked to choose the original faces of familiar people in a set of faces which varied in terms of identity strength, sex, or race information, they tended to select either anti-caricatures (in caricature/anti-caricature face sets) or faces with enhanced categorical information (in race and sex face sets). Specifically, more masculine (feminine) exemplars of familiar male (female) faces were chosen and more Asian (White) exemplars of familiar Asian (White) faces were chosen. Here, the results of Experiment 2 suggest that such interdependence of identity and sex information applies similarly to both familiar and unfamiliar faces and applies to both human and certain model-based face processing (e.g., AlexNet). Further research is needed to elucidate the mechanisms underlying this intriguing interdependence. It is worth mentioning that we found that average male and female faces were better sex-categorized than original faces of male and female people (unpublished results; see^19^), which is consistent with our results here.

Note that our department group should be able to recognize those modified personally familiar faces. For the sex-changed faces, some participants reported that they had noticed that some of the familiar female faces had got a beard, suggesting that familiar faces remain identifiable even under the disguise of a sex manipulation. Participants may get access to the original sex information of these sex-changed familiar faces via identity analysis, as the original sex information is visually absent in the stimuli but is bound to the identity of the face. This observation is consistent with our previous study^20^, which showed that for very familiar faces, participants had difficulty in differentiating the original version from its sex-changed version. For the caricaturized faces, caricatures of familiar faces (i.e., with enhanced identity strength) are often beneficial for recognition^40–42^. Caricatures often produce equivalent or better recognition performance relative to recognition of the original faces^43–47^. Anti-caricatures that reduce the strength of face identity, however, are reported to be less recognizable than the originals^44,47,48^ but are often chosen as better likeness^20,49,50^. For instance, it has been shown that participants are only able to attain 55.5% identification accuracy for the 50% level anti-caricatures of famous faces (i.e., halfway between the original and the average face)^44^. Leopold et al.^36^ have shown that faces with about 11% identity strength can still lead to 50% accuracy in recognition (see also^51^). The anti-caricatures used in the present study consist of 60% of the original face and 40% of the average face, which should remain largely recognizable.

By comparing model-based sex categorization with human performance, the present study not only offers some insights into how identity and sex are combined in human and model-based face perception but also highlights some of their crucial differences. For instance, while both our Experiment 1 and prior works have shown a consistent male bias in sex categorization^30–32^, our model-based sex classification does not exhibit such a bias. Similarly, while reducing identity strength (i.e., anti-caricatures) improved our participants’ sex categorization performance, it does not produce the same responses in the three models we used (Inception-ResNet even showed a reverse pattern of responses). In addition, although enhancing identity strength (i.e., caricaturizing faces) impaired sex categorization in both humans and models, it seems to have a stronger influence on the model-based face categorization than seen in human performance. Human categorization accuracy dropped by about 0.03 from originals to caricatures, whereas the accuracy of model classification was reduced remarkably (Alexnet, 0.33; Resnet, 0.15; Inception-ResNet, 0.26). Similar differences have also been observed in identity processing. Hancock et al.^15^ recently reported that many face recognition models based on deep neural networks produce recognition errors that humans rarely make. For instance, when the sex or race information of a face was changed, most models classified such modified faces as depicting the same person as the original face. These results suggest that humans and computational models do not rely on the same mechanism to recognize the sex or identity of a face.

While our study focuses on how sex, identity and familiarity jointly modulate sex categorization of faces, other face- or observer-based factors might also affect sex classification. The male bias in sex categorization is one example, whereas the influence of differing participants’ age on sex categorization accuracy did not occur (but see Supplementary Material Section B for its influence on response time). One limitation of the present study is that we did not eliminate all these potential face- and participants-based contributions to sex categorization, such as matching the ratio of male and female faces and their distinctiveness in the two sets of face stimuli and matching demographical information (e.g., age) across the two groups of participants. Although our main results are unlikely to be driven by these factors, these mismatches induced some seemingly surprising results, such as increased performance for the sex-changed unfamiliar faces (Experiment 1). In addition, although previous studies suggest that other invariant aspects of faces, such as race, may be similarly linked to the identity as the sex of faces is^9,10,14,20^, it remains to be elucidated exactly how race and identity may interact in humans and computational models. For instance, it has been shown that face recognition models using deep neural networks have different sensitivities to changes of sex or race in faces^15^. It is worth noting that our results are based on categorization of white faces by white participants, generalization of the current findings to other cultures should be made with caution.

In sum, the present study demonstrates that varying faces along either sex or identity dimension affects the perception of the sex of faces, irrespective of their familiarity status. When familiar faces are displayed with the appearance of the opposite sex, familiarity with faces impairs our ability to categorize their sex using exclusively visual facial features. When identity-defining information is enhanced or reduced in faces, perception of the sex of those faces also changes, with better performance for faces of reduced identity strength and worse performance for faces of enhanced identity strength. Moreover, modifying either the sex of a face or its identity strength impacted human performance and responses of neural network models differently. In addition, our face modification also affected responses of the three neural network models differently. These results not only provide further support for the view that the sex and identity of faces are integratively learned and processed^9,10,14^, but also reveal an intertwining of sex and identity dimensions in both human and model-based face perception.

### Supplementary Information

Supplementary Information.

## Data Availability

The data generated and analyzed in the two experiments reported in this study are available in the OSF repository (https://osf.io/4u5hw/?view_only=6d599379ac9b45cab5a110919763c080).

## References

[1] Bruce V, Young A (2012). Face Perception.

[2] Calder AJ, Rhodes G, Johnson MH, Haxby JV (2011). Oxford Handbook of Face Perception.

[3] Rapcsak SZ (2019). Face recognition. Curr. Neurol. Neurosci. Rep..

[4] Bruce V, Young AW (1986). Understanding face recognition. Br. J. Psychol..

[5] Bruce V, Ellis H, Gibling F, Young A (1987). Parallel processing of the sex and familiarity of faces. Can. J. Exp. Psychol..

[6] Ellis AW, Young AW, Flude BM (1990). Repetition priming and face processing: Priming occurs within the system that responds to the identity of a face. Q. J. Exp. Psychol..

[7] Goshen-Gottstein Y, Ganel T (2000). Repetition priming for familiar and unfamiliar faces in a sex-judgment task: Evidence for a common route for the processing of sex and identity. J. Exp. Psychol. Learn. Mem. Cogn..

[8] Rossion B (2002). Is sex categorization from faces really parallel to face recognition?. Vis. Cogn..

[9] Zhao M, Hayward WG (2013). Integrative processing of invariant aspects of faces: Effect of gender and race processing on identity analysis. J. Vis..

[10] Kramer RSS, Young AW, Day MG, Burton AM (2017). Robust social categorization emerges from learning the identities of very few faces. Psychol. Rev..

[11] Balas B, Cox DD, Conwell E (2007). The effect of real-world personal familiarity on the speed of face information processing. PLoS One.

[12] Ganel T, Goshen-Gottstein Y (2002). Perceptual integrality of sex and identity of faces: Further evidence for the single-route hypothesis. J. Exp. Psychol. Hum. Percept. Perform..

[13] Rakic T, Steffens MC, Wiese H (2018). Same-gender distractors are not so easy to reject: ERP evidence of gender categorization. Cogn. Affect. Behav. Neurosci..

[14] Dahl CD, Rasch MJ, Bülthoff I, Chen C-C (2016). Integration or separation in the processing of facial properties—A computational view. Sci. Rep..

[15] Hancock PJB, Somai RS, Mileva VR (2020). Convolutional neural net face recognition works in non-human-like ways. R. Soc. Open Sci..

[16] Minot T, Dury HL, Eguchi A, Humphreys GW, Stringer SM (2017). The neural representation of the gender of faces in the primate visual system: A computer modeling study. Psychol. Rev..

[17] Wallis G (2013). Toward a unified model of face and object recognition in the human visual system. Front. Psychol..

[18] Haxby JV, Gobbini MI, Furey ML, Ishai A, Schouten JL, Pietrini P (2001). Distributed and overlapping representations of faces and objects in ventral temporal cortex. Science.

[19] Bülthoff I, Newell FN (2004). Categorical perception of sex occurs in familiar but not unfamiliar faces. Vis. Cogn..

[20] Bülthoff I, Zhao M (2020). Personally familiar faces: Higher precision of memory for idiosyncratic than for categorical information. J. Exp. Psychol. Learn. Mem. Cogn..

[21] Lapuschkin, S., Binder, A., Muller, K.-R. & Samek, W. Understanding and comparing deep neural networks for age and gender classification. In *Proceedings of the IEEE International Conference on Computer Vision (ICCV)*, 1629–1638 (2017).

[22] Levi, G. & Hassner, T. Age and gender classification using convolutional neural networks. In *Proceedings of the IEEE Conference on Computer Vision and Pattern Recognition (CVPR) workshops*, 34–42 (2015).

[23] Krizhevsky, A., Sutskever, I. & Hinton, G. E. ImageNet classification with deep convolutional neural networks. In *Proceedings of International Conference on Neural Information Processing Systems (NIPS)*, 1097–1105 (2012).

[24] He, K., Zhang, X., Ren, S. & Sun, J. Deep residual learning for image recognition. In *Proceedings of the IEEE Conference on Computer Vision and Pattern Recognition*, 770–778 (2016).

[25] Szegedy, C., Ioffe, S., Vanhoucke. V. & Alemi, A. A. Inception-v4, inception-ResNet and the impact of residual connections on learning. In *Proceedings of the Thirty-First AAAI Conference on Artificial Intelligence*, 4278–4284 (2017).

[26] Rajalingham R, Issa EB, Bashivan P, Kar K, Schmidt K, DiCarlo J (2018). Large-scale, high-resolution comparison of the core visual object recognition behavior of humans, monkeys, and state-of-the-art deep artificial neural networks. J. Neurosci..

[27] Bruce V, Burton AM, Hanna E, Healey P, Mason O, Coombes A, Fright R, Linney A (1993). Sex discrimination: How do we tell the difference between male and female faces?. Perception.

[28] Blanz V, Vetter T (1999). A morphable model for the synthesis of 3D faces. Proceedings of the 26th annual conference on Computer Graphics and Interactive Techniques—SIGGRAPH ’99.

[29] Troje NF, Bülthoff HH (1996). Face recognition under varying poses: The role of texture and shape. Vis. Res..

[30] Armann R, Bülthoff I (2012). Male and female faces are only perceived categorically when linked to familiar identities—And when in doubt, he is a male. Vis. Res..

[31] Brielmann AA, Gaetano J, Stolarova M (2015). Man, you might look like a woman—If a child is next to you. Adv. Cogn. Psychol..

[32] Wild HA, Barrett SE, Spence MJ, O’Toole AJ, Cheng YD, Brooke J (2000). Recognition and sex categorization of adults’ and children’s faces: Examining performance in the absence of sex-stereotyped cues. J. Exp. Child Psychol..

[33] Davidenko N (2007). Silhouetted face profiles: A new methodology for face perception research. J. Vis..

[34] Gaetano J, Van Der Zwan R, Oxner M, Hayward WG, Doring N, Blair D, Brooks A (2016). Converging evidence of ubiquitous male bias in human sex perception. PLoS One.

[35] Graf AB, Wichmann FA, Bülthoff HH, Lee S-W, Poggio TA, Wallraven C (2002). Gender classification of human faces. Biologically Motivated Computer Vision. Lecture Notes in Computer Science.

[36] Leopold DA, O’Toole AJ, Vetter T, Blanz V (2001). Prototype-referenced shape encoding revealed by high-level aftereffects. Nat. Neurosci..

[37] O’Toole AJ, Vetter T, Volz H, Salter EM (1997). Three-dimensional caricatures of human heads: Distinctiveness and the perception of facial age. Perception.

[38] Valentine T, Lewis MB, Hills PJ (2016). Face-space: A unifying concept in face recognition research. Q. J. Exp. Psychol..

[39] Stroop JR (1935). Studies of interference in serial verbal reactions. J. Exp. Psychol..

[40] Kaufmann JM, Schulz C, Schweinberger SR (2013). High and low performers differ in the use of shape information for face recognition. Neuropsychologia.

[41] Kaufmann JM, Schweinberger SR (2012). The faces you remember: Caricaturing shape facilitates brain processes reflecting the acquisition of new face representations. Biol. Psychol..

[42] Mauro R, Kubovy M (1992). Caricature and face recognition. Mem. Cognit..

[43] Chang PPWW, Levine SC, Benson PJ (2002). Children’s recognition of caricatures. Dev. Psychol..

[44] Lee K, Byatt G, Rhodes G (2000). Caricature effects, distinctiveness, and identification: Testing the face-space framework. Psychol. Sci..

[45] Lee KJ, Perrett DI (2000). Manipulation of colour and shape information and its consequence upon recognition and best-likeness judgments. Perception.

[46] Rhodes G, Byatt G, Tremewan T, Kennedy A (1997). Facial distinctiveness and the power of caricatures. Perception.

[47] Rhodes G, Brennan SE, Carey S (1987). Identification and ratings of caricatures: Implications for mental representations of faces. Cogn. Psychol..

[48] Stevenage SV (1995). Can caricatures really produce distinctiveness effects?. Br. J. Psychol..

[49] Allen H, Brady N, Tredoux C (2009). Perception of “best likeness” to highly familiar faces of self and friend. Perception.

[50] Hancock PJB, Little AC (2011). Adaptation may cause some of the face caricature effect. Perception.

[51] Bülthoff I, Zhao M (2021). Average faces: How does averaging process change faces physically and perceptually?. Cognition.

