# Supplementary material for: Varying sex and identity of faces affects face categorization differently in humans and computational models
Source: Sci Rep. 2023 Sep 26;13:16120. doi: 10.1038/s41598-023-43169-9 (PMC10522766; doi:10.1038/s41598-023-43169-9)
Supplement: Supplementary file 1 — Supplementary Information. [file 41598_2023_43169_MOESM1_ESM.docx]

**Varying sex and identity of faces affects face categorization differently**

**in humans and computational models**

Isabelle Bülthoff, Laura Manno, and Mintao Zhao

**Supplementary Material**

# Section A

**Results of omnibus ANOVAs for Experiments 1 and 2**

**1. Experiment 1: Accuracy**

Mean accuracy data of Experiment 1 are shown in **Figure 2**. The accuracy data were submitted to a mixed 2 X 2 X 2 ANOVAs, with *face set* (colleague set vs unfamiliar set) and *face manipulation* (original vs sex-changed) as within-participants factors and *participants group* (department vs control group) as between-participants factor. All three main effects in the ANOVAs were significant. Sex categorization performance was better for original faces (0.810±0.016) than for sex-changed faces (0.755±0.014), *F*(1,45) = 7.210, *p* = .010; *ηp*^2^ = .138. Unfamiliar face set (0.807±0.013) elicited an overall better performance than that for the colleague face set (0.758±0.013), *F*(1,45) = 16.358, *p* < .001; *ηp*^2^ = .267. Participants in the control group (0.810±0.016) showed higher performance than participants in the department group (0.758±0.016), *F*(1,45) = 4.687, *p* = .036; *ηp*^2^ = .094.

The three significant main effects were modulated by significant interactions between face manipulation and face set, *F*(1,45) = 95.106, *p* < .001; *ηp*^2^ = .679, and between face manipulation and participants group, *F*(1,45) = 10.218, *p* = .003; *ηp*^2^ = .185. None of other effects was significant, all *F*(1,45) ≤ 0.450, *p* ≥ .506; *ηp*^2^ ≤ .010.

**2. Experiment 1: Response time**

Mean RT data of Experiment 1 are shown in **Figure 3**. We performed the same ANOVAs as we did for accuracy data. The results showed that participants took longer to categorize the sex-changed faces (926±43 ms) than to categorize the original faces (882±35 ms), revealing a significant main effect of face manipulation, *F*(1,45) = 5.456, *p* = .024; *ηp*^2^ = .108;. Participants in the control group responded more quickly (812±54 ms) than those in the department group (995±53 ms), *F*(1,45) = 5.824, *p* = .020; *ηp*^2^ = .115. The ANOVAs also showed significant interactions between face manipulation and face set, *F*(1,45) = 16.044, *p* < .001; *ηp*^2^ = .263, and between face manipulation and participants group, *F*(1,45) = 10.046, *p* = .003; *ηp*^2^ = .183. None of other effects was significant, all *F*(1,45) ≤ 2.982, *p* ≥ .091; *ηp*^2^ ≤ .062.

**3. Experiment 2: Accuracy**

Mean accuracy data of Experiment 2 are shown in **Figure 6**. The data were submitted to a mixed 2 X 3 X 2 ANOVAs, with two within-participants factors (face set: *colleague*, *unfamiliar*; face manipulation: anti-caricature, original, caricature) and one between-participants factor (participant group: department vs control group). We found a significant main effect of face manipulation, *F*(2,76)= 34.002, *p* <.001, *ηp^2^* = . 472. Within-subject contrasts showed that sex categorization performance was higher for anti-caricatures (0.957±0.008) than for original faces (0.909±0.013), *F*(1,38) = 22.783, *p* <.001, *ηp*^2^ = . 375, which was higher than that for caricature faces (0.863±0.014), *F*(1,38) = 15.323, *p* <.001, *ηp*^2^ = .287. This ordered pattern of response was similar for both sets of faces and for both groups of participants; none of the interactions involving face manipulation was significant, all *F*s ≤ 1.174; all *p*s ≥ .315, all *ηp*^2^ ≤ .030. The overall performance was similar between the two groups of participants, *F*(1,38)= 0.002, *p* =.965, *ηp*^2^ < .001, and between the two sets of faces, *F*(1,38)= 1.131, *p* =.294, *ηp*^2^ = .029. However, the interaction between participants group and face set was significant, *F*(2,76)= 8.880, *p* =.005, *ηp*^2^ = .189.

**4. Experiment 2: Response time**

Mean response time data are shown in **Figure 7**. The same 2 X 3 X 2 ANOVAs revealed a significant main effect of face manipulation, *F*(2,76)= 9.946, *p* <.001, *ηp*^2^ = .207. Within-subject contrasts showed that participants took longer for categorizing caricatures (900±37 ms) than for categorizing original faces (847±29 ms), *F*(1,38) = 9.547, *p* =.004, *ηp*^2^ = .201, and anti-caricature faces (827±32 ms), *F*(1,38) = 13.020, *p* <.001, *ηp*^2^ = .255]. The latter two conditions showed no significant difference, *F*(1,38) = 2.460, *p* =.125, *ηp*^2^ = .061. This pattern of response was similar for both sets of faces and for both groups of participants; as none of the interactions involving face manipulation was significant, all *F*s ≤ 2.881; all *p*s ≥ .062, all *ηp*^2^ ≤ .070.

We also found a significant main effect of face set, *F*(1,38)= 5.258, *p* =.027, *ηp*^2^ = .122. Participants responded faster to the colleague face set (842±31 ms) than to the unfamiliar face set (874±33 ms). The main effect of participants group did not reach statistical significance, *F*(1,38) = 3.282, *p* =.078, *ηp*^2^ = .079. However, the interaction between face set and participants group was significant, *F*(1,38) = 10.731, *p* =.002, *ηp*^2^ = .220.

# Section B

**Results of Response Time for Experiments 1 and 2**

**1. Experiment 1**

**Figure S1** shows the results of response time in Experiment 1. For the *unfamiliar face set*, A 2 (participants group) by 2 (face manipulation) ANOVAs revealed a significant main effect of participants group, *F*(1,45) = 18.625, *p* < .001; *ηp*^2^ = .293. The control group (805±57 ms) responded faster than the department group (1000±56 ms). Neither the main effect of face manipulation, *F*(1,45) = 0.556, *p* = .460; *ηp*^2^ = .012, nor its interaction with participants group, *F*(1,45) = 1.854, *p* = .180; *ηp*^2^ = .040, was significant. These results indicate that the responses of both groups were not affected by face manipulation.

For the *colleague face set*, the ANOVAs revealed significant main effects of face manipulation, *F*(1,45) = 18.625, *p* < .001; *ηp*^2^ = .293, and participants group, *F*(1,45) = 5.275, *p* = .026; *ηp*^2^ = .105. Responses were faster to the original faces than to the sex-changed faces (852±33 ms vs 958±45 ms) and were faster in the control group than in the department group (819±54 ms vs 991±52 ms). Importantly, the interaction between face manipulation and participants group was significant, *F*(1,45) = 12.379, *p* = .001; *ηp*^2^ = .216. Follow-up paired t-test showed that while both groups had equivalent performance on the original faces, *t*(45) = 1.314, *p* (uncorrected) = .195, Cohen’s *d* = 0.384, the department group was significantly slow in responses than the control group, *t*(45) = 2.856, *p* (uncorrected) = .006, Cohen’s *d* = 0.833. Consistent with the accuracy data, these results indicate that sex manipulation affected performance of both groups differently; it had a stronger impact on the department group – who was familiar with the original faces of the colleague set – than on the control group.


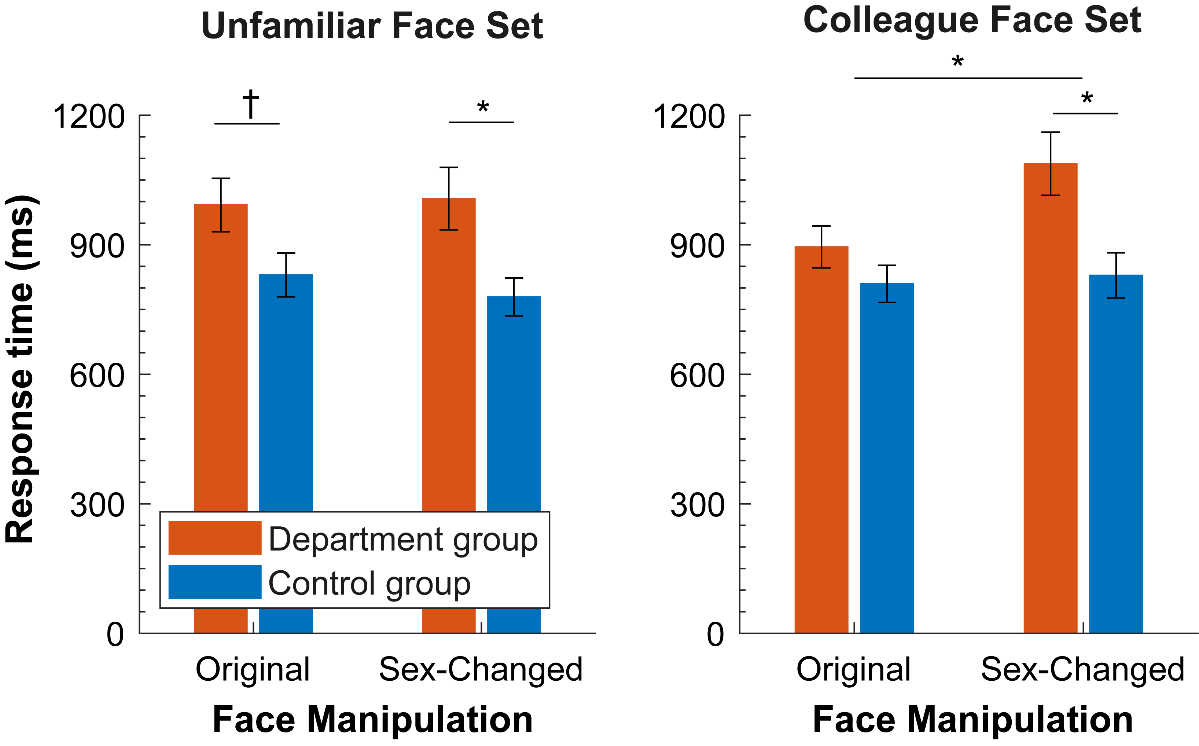


**Figure S1**. Mean response time data in Experiment 1 for each face set. Long horizontal bars indicate significance of interaction, and short bars indicate significance of follow-up contrast. *, *p* ≤ .01; †, *p* =.05. Error bars represent SEMs.

Our control group showed quicker responses than the department group. This may be due to their differences in age (mean age for the control and department group are 27 and 34 years respectively). Correlation analyses between participants’ age and their response times for each face condition showed a consistent trend for younger participants to respond faster (see **Correlation analyses** below and **Figure S3** for detailed results).

**2. Experiment 2**

Mean response time data are shown in **Figure S2**.


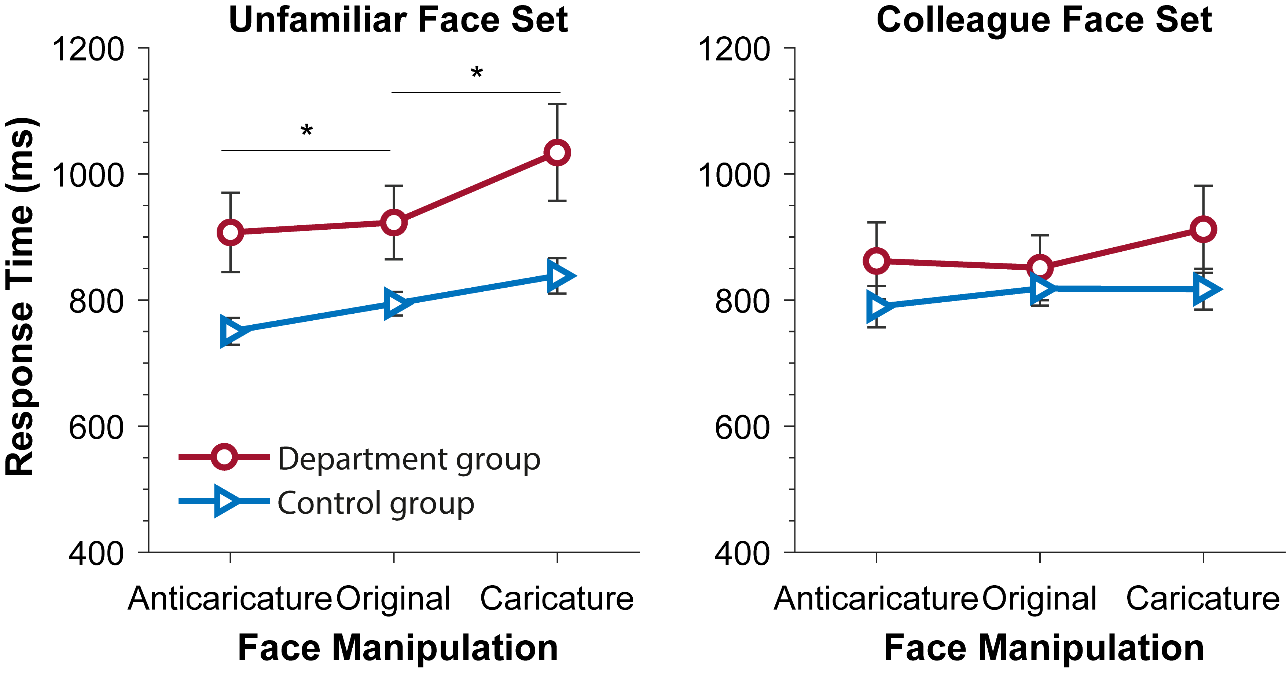


**Figure S2**. Mean response time data in Experiment 2 for each face set. Horizontal bars indicate significance of difference between face manipulation conditions. *, *p* < .05. Error bars represent SEMs.

For the *unfamiliar face set,* a 3 (face manipulation: anti-caricature, original, caricature) by 2 (group: unfamiliar vs department group) ANOVA revealed a main effect of face manipulation, *F*(2, 76) = 14.006, *p* < .001, *ηp*^2^ = .269. Within-participants contrasts showed that sex categorization performance was numerically faster for anti-caricatures (829±33 ms) than for original faces (858±31 ms), *F*(1,38) = 3.485, *p* =.070, *ηp*^2^ = .084, which was significantly faster than that for caricature faces (936±41 ms), *F*(1,38) = 11.732, *p* < .001, *ηp*^2^ = .236. Participants in the department group responded more slowly than the control group, *F*(1,38) = 5.910, *p* =.020, *ηp*^2^ = .135, but the interaction between participants group and face manipulation was not significant, *F*(2,76) = 1.278, *p* =.282, *ηp*^2^ = .033. These results indicate that increasing face identity strength prolongs participants reaction time in sex categorization.

For *the colleague face set,* the same ANOVA revealed no significant results. Neither the main effects of face manipulation, *F*(2, 76) = 1.472, *p* = .236, *ηp*^2^ = .037, participants group, *F*(2, 76) = 1.139, *p* = .293, *ηp*^2^ = .029, nor their interaction, *F*(2,76) = 0.873, *p* =.401, *ηp*^2^ = .022, was significant. These results suggest that familiarity with the original identity of the faces does not speed up sex categorization of those faces.

As in Experiment 1, differences in participants’ average age may account for the overall quicker response of the control group compared to the department group. Consistent with this speculation, we found significant between participants’ age and their response times for each face condition: younger participants often responded quicker correlations (see **Correlation analyses** below and **Figure S4** for detailed results**)**.

**3. Correlation analysis between participants’ age and their performance**

As our control group showed quick responses than our department group, and the control group also on average younger than the department group, we performed correlation analysis to see if participants’ age affects their response time. We also performed the same correlation analysis between age and response accuracy for completeness. The results for Experiment 1 are shown in Figure S3, and the results for Experiment 2 are shown in Figure S4.

Altogether, correlation analyses between participants’ age and performance in both experiments do not show any constant influence of participants’ age onto response accuracy, whereas the younger control group always tended to respond faster than the department group.


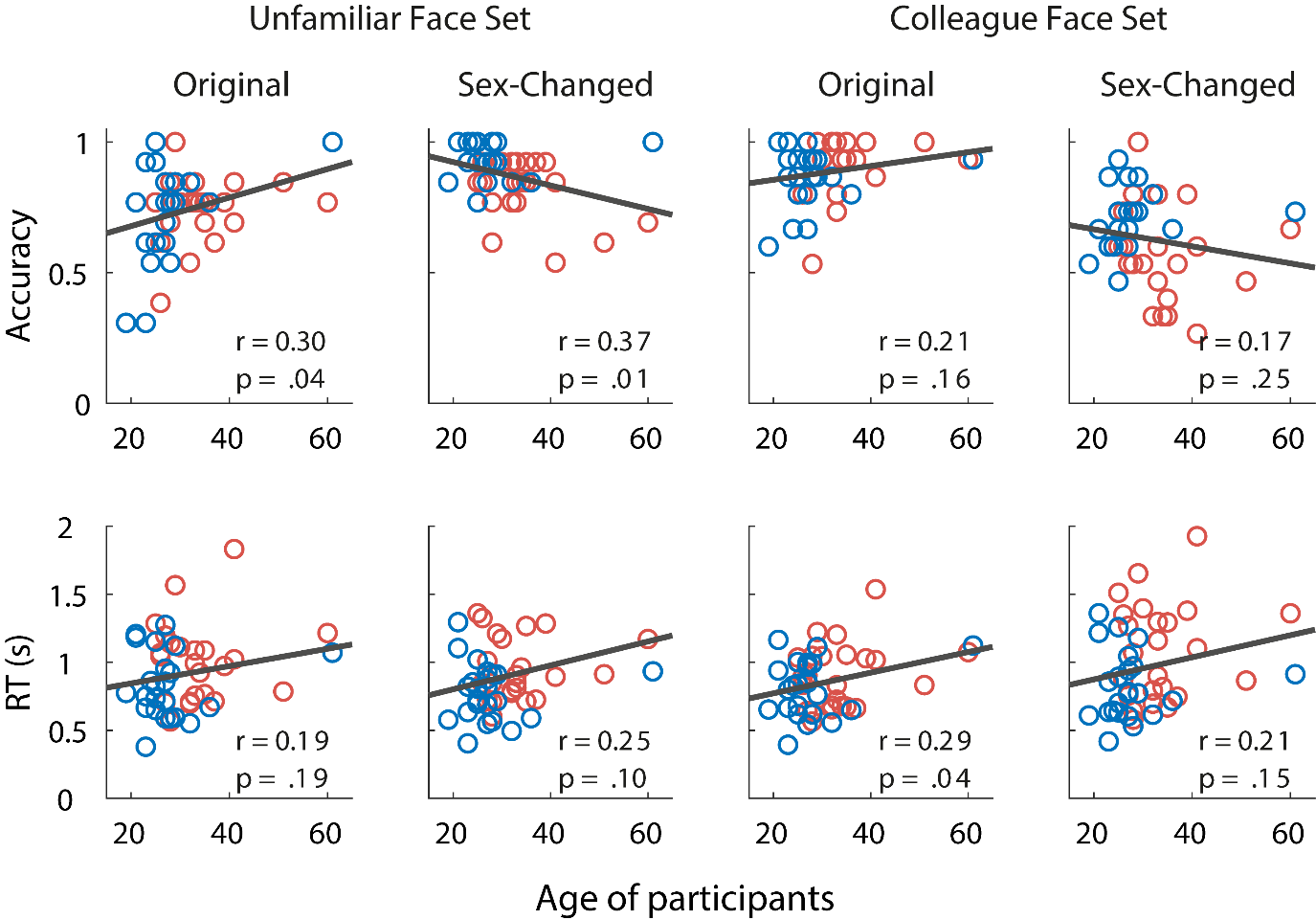


**Figure S3**. Results of correlation analysis between the age of participants and their responses in Experiment 1. Upper panel: participants showed a mixture trend of linear relationship between their age and their response accuracy. Lower panel: participants showed consistent trend of increasing response time with increasing age. Data points represents individual participants, with the control group shown in blue color and the department group shown in red color.


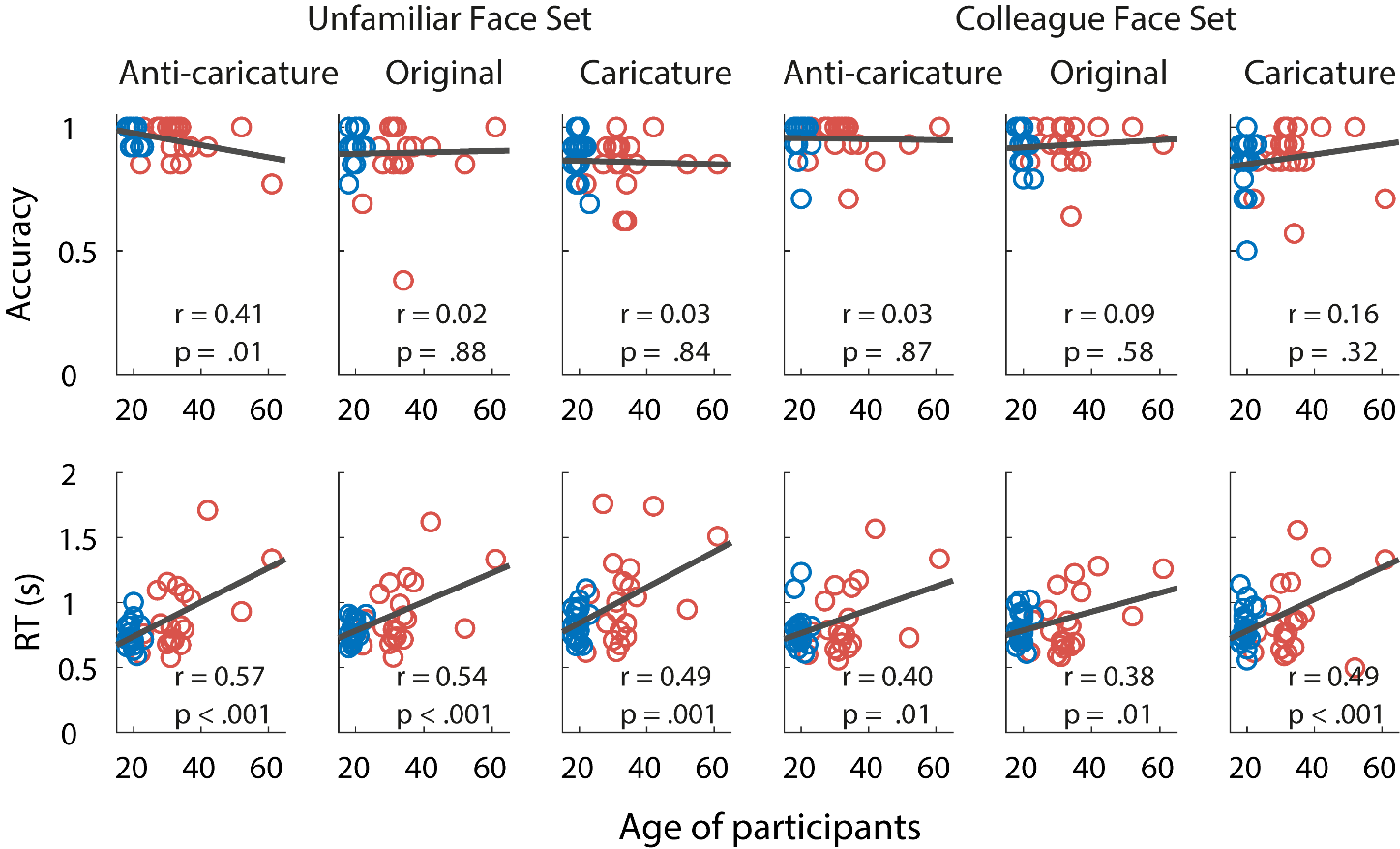


**Figure S4**. Results of correlation analysis between the age of participants and their responses in Experiment 2. Upper panel: participants’ response accuracy was generally not related to their age except for the anti-caricatures, which showed decreasing accuracy with increasing age. Lower panel: participants showed consistent significant correlations between their age and their response time. Across all face conditions, response times increased with increasing age. Data points represents individual participants, with the control group shown in blue color and the department group shown in red color.

# Section C

**Model-Based Sex Categorization Measured Using Response Probability**

**1. Experiment 1**

As shown in **Figure S5**, all three models showed patterns of responses differing from human data. For AlexNet responses, none of the main effects or their interaction were significant, all *F*s < 1.007, all *p*s ≥ .325, all ηp^2^ ≤ .037. The model responded similarly to both sets of faces (colleague face set: 0.829±0.030, unfamiliar face set: 0.832±0.032), and showed no difference between original faces (0.831±0.045) and sex-changed faces (0.830±0.038). These results suggest that sex-manipulation does not affect the model’s sex-categorization performance.

For responses of the ResNet, we only found a significant main effect of face manipulation, *F*(1,26) = 13.956, *p* < .001; *ηp*^2^ = .349. Sex categorization was better for original faces (0.874±0.040) than for sex-changed faces (0.480±0.076). The main effect of face set and its interaction with face manipulation were not significant, both *F*s < 1, *p*s ≥ .417, both *ηp*^2^ ≤ .025. Thus, changing the sex of faces impacted sex categorization of the ResNet model similarly for both face sets.

For responses of the Inception-ResNet, both the main effect of face manipulation, *F*(1,26) = 28.650, *p* < .001; *ηp*^2^ = .524, and face set, *F*(1,26) = 5.403, *p* = .028; *ηp*^2^ = .172, were significant. Their interaction was not significant, *F*(1,26) = 2.494, *p* = .126; *ηp*^2^ = .088. Therefore, changing sex of faces impacted sex-categorization of the Inception-ResNet model for both sets of faces.

**
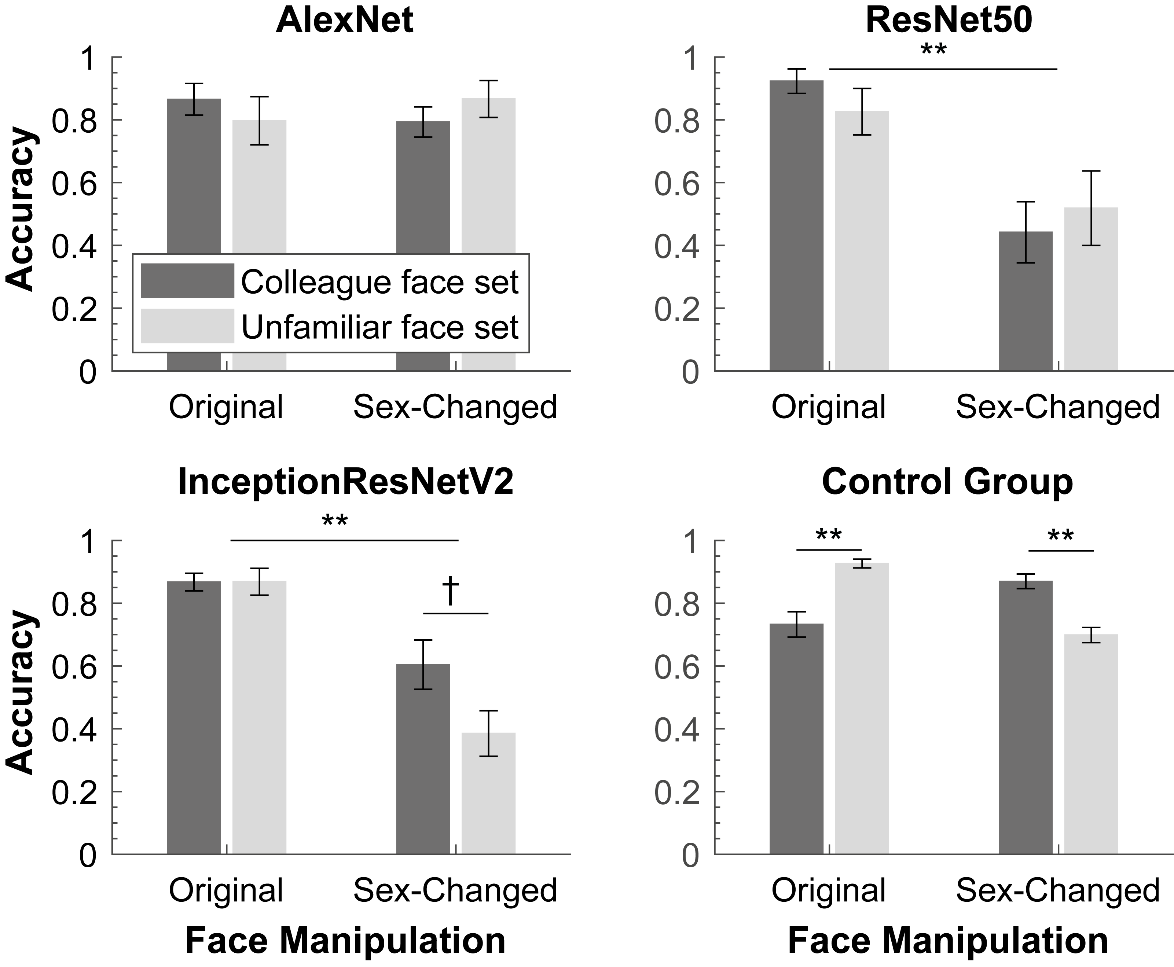
**

**Figure S5**. Model-based sex categorization performance measured using response probability in Experiment 1. The results of the control group are replotted here to show the pattern of human performance. Long horizontal bars indicate significance of face manipulation effect, and short bars indicate significance of follow-up contrast. **, *p* < .001; †, *p* = .053. Error bars represent SEMs.

**2. Experiment 2**

As shown in **Figure S6**, the three models responded differently to the sex-categorization task, and only the AlexNet model showed a pattern of response similar to that observed with our control group. For AlexNet responses, The main effect of face manipulation was significant, *F*(2,50) = 15.352, *p* < .001, *ηp*^2^ = .380. Sex categorization performance was higher for anti-caricatures (0.945±0.018) than for original faces (0.816±0.046), *F*(1,25) = 7.237, *p* = .013, *ηp*^2^ = .224, and performance for original faces was in turn higher than that for caricatures (0.596±0.081), *F*(1,25) = 19.317, *p* < .001, *ηp*^2^ = .436. The main effect of face set and the interaction between face set and face manipulation were not significant, both *F* < 1. This ordered pattern of responses was similar to human performance, though increasing identity strength seemed to have a stronger effect on model responses than on human performance.

For responses of the ResNet50, there was only a marginally significant main effect of face manipulation, *F*(2,50) = 3.087, *p* = .054, *ηp*^2^ = .110. Follow-up contrast indicate that this effect is driven by higher categorization performance for the original faces (0.912±0.038) than for the caricatures (0.737±0.071), *F*(1,25) = 9.651, *p* = .005, *ηp*^2^ = .279. No significant difference was found when the above two conditions were compared to performance on anticarictures (0.880±0.047), both *F*s ≤ 2.116, *p*s ≥ .158, both *ηp*^2^ ≤ .078. Thus, manipulation of face identity strength does not show a strong influence on sex-categorization of the ResNet50 model.

For responses of the Inception-ResNet-V2, neither of the two main effects nor their interaction was significant, all *F*s ≤ 1.734, *p*s ≥ .187, all *ηp*^2^ ≤ .065. Therefore, sex-categorization of this model is not affected by the change of identity strength and showed no difference between the two face sets.


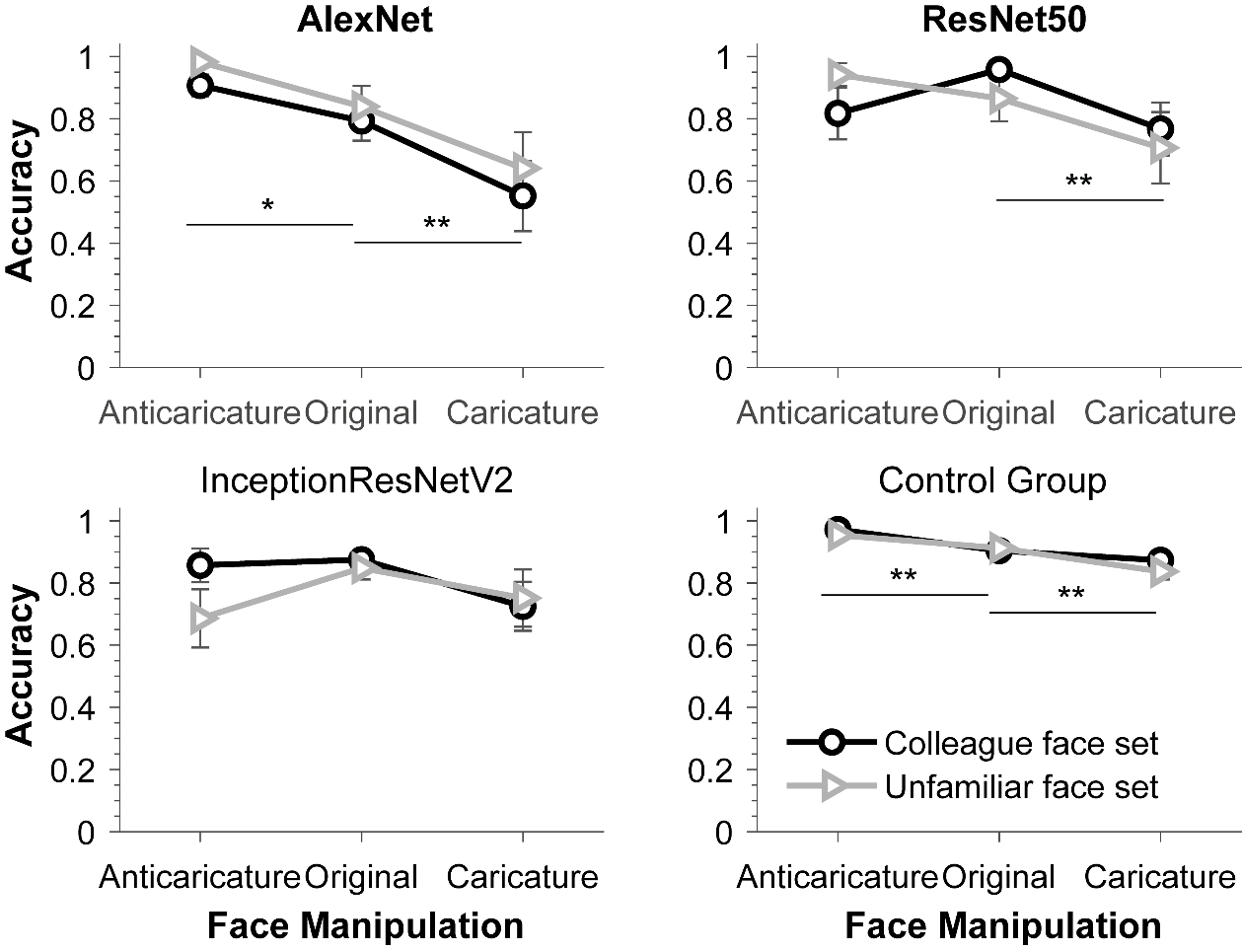


**Figure S6**. Model-based sex categorization performance measured with response probability in Experiment 2. The results of the control group are replotted here to show the human response pattern. Horizontal bars indicate significance of difference following a main effect of face manipulation. **, *p* ≤ .005; *, *p* < .05; Error bars represent SEMs.
